# Comparative Genomics and Phylogenetic Analyses of *Aquarius macrophyllus* and Related Genera in Alismataceae Based on Plastome Data

**DOI:** 10.1002/ece3.71568

**Published:** 2025-06-10

**Authors:** Jie Li, Shengnan Wei, Jianan Ying, Qixiang Lu

**Affiliations:** ^1^ Zhejiang Province Key Laboratory of Plant Secondary Metabolism and Regulation College of Life Sciences and Medicine, Zhejiang Sci‐Tech University Hangzhou China

**Keywords:** Alismataceae, *Aquarius macrophyllus*, comparative genomics, phylogenetics, plastome

## Abstract

*Aquarius macrophyllus*, an aquatic species native to South America, has been widely utilized as an ornamental plant. Although the plastome data have been commonly used in plant evolution and phylogenetics, research on the plastome of *A. macrophyllus* remains scarce. In this study, we sequenced the plastome of *A. macrophyllus* and conducted a comparative analysis incorporating eight species from related genera of *Caldesia*, *Sagittaria*, *Alisma*, and *Luronium*. The assembled plastome was 180,860 bp in size, with 133 genes annotated, including 88 protein‐coding genes, 8 rRNA, and 37 tRNA. Comparative analyses showed that the genome lengths of the nine plastomes ranged from 159,063 to 180,860 bp, including a large single copy (LSC, 89,203–99,125 bp), a small single copy (SSC, 10,131–19,753 bp), and a pair of inverted repeat sequences (IR, 25,124–39,664 bp). The nine plastomes were similar in GC content, codon usage, and gene distribution but contained variations, including IR expansion in *A. macrophyllus* , loss of *trnV‐UAC* introns, and gene inversion in *Caldesia and Sagittaria* species. Two sequence inversions of 5.6 and 6.4 kb were detected in *Sagittaria* and *Caldesia*, respectively. A total of 532 simple sequence repeats (SSRs) and 667 dispersed repeats were detected in the nine plastomes. Selection pressure analysis using *A. macrophyllus* as a reference to compare with other species revealed that three ribosomal protein genes *rps2*, *rps18*, and *rps7* were positively selected, which showed intergeneric specificity, especially displaying higher selection pressure in *Sagittaria* species than that in *Caldesia* species. Phylogenetic analysis of Alismataceae elucidated that *Alisma*, *Luronium*, and *Burnatia* species were early diverged, followed by *Hydrocleys* and *Limnocharis*. The monophyly of *Aquarius* and its sister relationship with *Echinodorus* were revealed, supporting the classification of the new genus *Aquarius*. Our study enriches the genomic resources of *Aquarius* and provides new insights into plastome structure and evolution in Alismataceae.

## Graphical abstract

We sequenced the plastome of *Aquarius macrophyllus* and conducted a comparative analysis incorporating eight species from four related genera in Alismataceae. Phylogenetic analysis of Alismataceae elucidated that Alisma and Burnatia species were early diverged, followed by Hydrocleys and Limnocharis. The monophyly of Aquarius and its sister relationship with Echinodorus were revealed, supporting the classification of the new genus Aquarius.

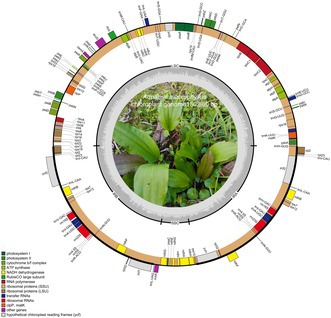

## 1 Introduction

The family Alismataceae, one of the oldest lineages of monocotyledons (Chen et al. 2012), comprises 19 genera and 90–100 species, including aquatic or semi‐aquatic herbs with subcosmopolitan distribution (Chen et al. 2012; Li et al. 2022; The Angiosperm Phylogeny Group 2016). Most species of Alismataceae have emergent or floating leaves, displaying a variety of life forms. *Echinodorus* is the second largest genus in the family Alismataceae (Ferreira et al. 2018), and its classification at the species level is constantly being updated. Currently, 26 *Echinodorus* species except *Echinodorus berteroi* previously attributed to *Echinodorus* are now classified as genus *Aquarius* (Govaerts et al. 2021; Plants of the World Online (POWO) 2023). *Aquarius macrophyllus*, synonymized as *Echinodorus macrophyllus*, is native to Guyana, western Brazil, and Argentina and has been introduced to other countries as a horticultural plant. For its broad leaves and immaculate white flowers, *Aquarius* is commonly used in aquatic plant landscapes. Previous studies of Brazilian *Echinodorus* (now *Aquarius*) and *Sagittaria* species were focused on their morphology and lifeform in semi‐arid climates and intermittent aquatic ecosystems (Matias 2007, 2010). Being rich in bioactive components, such as flavonoids (Ferreira et al. 2018; Fernandes et al. 2022), steroids and triterpenoids, *Aquarius macrophyllus* is also used as a folk medicine in Brazil for the treatment of inflammation, rheumatism, arthritis and kidney damage (Fernandes et al. 2022; da Silva et al. 2024; Tanus‐Rangel et al. 2010). Like most aquatic plants, as food for aquatic animals and substrate stabilizers, they are one of the primary producers in the underwater environment and are often found in aquariums (Rataj 1970). Furthermore, several studies have reported that Alismataceae species can be used for river restoration in phytoecological remediation (Handajani et al. 2018; da Silva et al. 2016). Due to the above application value, further genomic study of *A. macrophyllus* species would deepen the knowledge of *Aquarius* species classification and provide valuable information to understand the evolution of Alismataceae plants.

The plastome, plastid genome of plants structured as a circular DNA molecule with a length of approximately 120–210 kb and uniparentally inherited in most species, has been widely applied in resolving phylogenetic relationships in plant taxonomic groups (Guan et al. 2024). Comparative analysis of plastomes provides valuable information for genomic variation and effectively helps to reveal the process of plant evolution and diversification (Wu, Fan, et al. 2024; Cao et al. 2024). In general, plant plastomes are always conserved and consist of four parts: the large single‐copy region (LSC), the small single‐copy region (SSC), and two almost identical inverted repeat sequences (IRa and IRb). However, at higher plant taxa levels, sequence rearrangements or inversions in the plastomes have been reported in different plants (Kim et al. 2005; Liao et al. 2020), such as in Plantaginaceae, Lentibulariaceae, and Selaginellaceae (Xie et al. 2023; Silva et al. 2019; Xu et al. 2018). Different types of rearrangements result from changes in gene order and content in plastomes, including translocation, inversion, gene or intron deletion, gene duplication, and IR contraction/expansion (Liu et al. 2019; Chen, Zhang, et al. 2022). For instance, the expansion of the IR region in *Thalassia hemprichii* (Hydrocharitaceae) resulted in a reduction of the SSC region in the plastid genome (Chen, Zang, et al. 2023). These characteristics can be used in phylogenetic and evolutionary studies to understand interspecies relationships of plants (Shi, Hu, et al. 2023).

Until now, nearly 13,000 plastomes have been published, most of which are angiosperms, with more dicotyledons than monocotyledons (Wang, Kan, et al. 2024). Several plastome sequences of Alismataceae plants have been reported in recent years (Mwanzia et al. 2019; Zheng et al. 2024), but most of them are coding sequences (CDS) data. Most of the phylogenetic analyses of *A. macrophyllus* and Alismataceae were based on DNA fragments (ITS, *trnK* and *rbcL*, *matK* coding regions, etc.) and morphological data (Chen et al. 2012; The Angiosperm Phylogeny Group 2016; Lehtonen 2006; Lehtonen and Myllys 2008). However, due to the lack of sufficient polymorphic sites, several plastid loci may not represent the true species relationship. In addition, comparative analyses of the plastomes in Alismataceae are rarely performed. Currently, complete plastomes of *Aquarius* are limited, compared with other genera, such as *Sagittaria* and *Alisma*. Although the plastome of *Aquarius grisebachii* has been published in NCBI (https://www.ncbi.nlm.nih.gov/nuccore/PP061022.1), the IR region was asymmetric and its IRa appears to be incomplete, in addition to having only 124 annotated genes, which is fewer than the 133 genes of *A. macrophyllus* and the other species studied. These differences may introduce bias or error in the phylogenetic analysis, so we excluded *A. grisebachii* to maintain the accuracy of the phylogenetic analysis. For the two species of *Aquarius cordifolius* and *Aquarius paniculatus*, only CDS data were available (Chen et al. 2012; Luo et al. 2016), thus they were only used in phylogenetic analysis in our study. In order to study the chloroplast genome characteristics and systematic evolution of *A. macrophyllus* in the context of Alismataceae, we sequenced its complete plastome and conducted a comparative genomics analysis incorporating eight species from four genera of *Caldesia*, *Sagittaria*, *Alisma*, and *Luronium*. The aims of the study were as follows: (1) to investigate the plastome structure of *A. macrophyllus* and the variation compared with *Caldesia*, *Sagittaria*, *Alisma*, and *Luronium* at a genus level; (2) to screen highly variable hotspot regions for future population genetics; (3) to detect selection pressure on CDS genes; and (4) to resolve the phylogenetic relationship of *A. macrophyllus* within Alismataceae.

## 2 Materials and Methods

### 2.1 DNA Extraction and Sequencing

With permission, samples for this study were obtained from introduced plants at Hangzhou Botanical Garden (120°06′42.52″ E, 30°15′07.73″ N), Hangzhou, Zhejiang Province, China. The leaves were collected and dried with silica gel. The voucher specimens (No. QL22103003) were identified by Qixiang Lu and deposited in the Zhejiang University Herbarium (HZU, Hangzhou, China). Total genomic DNA was extracted from dried leaf samples using the CTAB method and the DNA Plantzol kit (Invitrogen, Carlsbad, CA, USA) (Chen, Wang, et al. 2022; Wang et al. 2021). The extraction process was performed according to the manufacturer's protocol. After assessing the quality and concentration of DNA using agarose gel electrophoresis, paired‐end (PE) sequencing libraries were constructed according to Illumina standard protocols (Illumina, San Diego, CA, USA) (Feng et al. 2022; Yin et al. 2022). Sequencing was performed on the Illumina HiSeq2500 platform at the Beijing Genomics Institute (BGI, Shenzhen, China).

### 2.2 Genome Assembly and Annotation

Complete plastid genomes were assembled using raw reads through de novo assembly and reference guidance (Zeng et al. 2021; Guo et al. 2023). Initially, we assembled the complete plastome of *A. macrophyllus* using the default parameters of NOVOPlasty v4.2.1 (Dierckxsens et al. 2017) and GetOrganelle v1.6.0 (Jin et al. 2020), with the reference plastome of *Sagittaria trifolia* (NC_044119) as a guide. Subsequently, the quadripartite structure was validated and examined using Bandage (Wick et al. 2015) and compared with the published plastomes of related species. Annotation was accomplished using GeSeq (Tillich et al. 2017) and Geneious Prime 2023.2.1 software (Kearse et al. 2012), with the reference genome *S. trifolia* (GenBank accession no. NC_044119). GB2Sequin (Lehwark and Greiner 2019) and CPGView (Liu et al. 2023) were used to confirm the annotation results and to check the accuracy of introns and trans‐spliced genes. Plastome map visualization was performed using the online software OGDraw (https://chlorobox.mpimp‐golm.mpg.de/OGDraw.html, February 29, 2024). After the assembly and annotation were completed, the sequence of *A. macrophyllus* was submitted to the GenBank database under accession number PP503046.

### 2.3 Plastome of *A. macrophyllus* and Comparative Genomics

A comparative genomic analysis was performed on nine species representing five genera within Alismataceae: *Sagittaria* ( *S. graminea* , *S. lichuanensis*, and *S. trifolia* ), *Caldesia* ( *C. grandis* and *C. parnassifolia* ), *Alisma* ( *A. plantago‐aquatica* and *A. canaliculatum* ), and *L. natans* , with *A. macrophyllus* as the reference genome. To investigate structural variations in plastid genomes, we analyzed the expansion and contraction of IR regions and visualized the structure of the IR/SSC and IR/LSC boundaries using the online tool CPJSdraw (Li, Guo, et al. 2023). In Shuffle‐LAGAN mode, the whole plastid genome of *A. macrophyllus* was compared with eight species using mVISTA (Frazer et al. 2004). Meanwhile, the nucleotide diversity (Pi) of the gene and spacer region sequences was calculated using DNASP v5.10 software (Librado and Rozas 2009), and the nucleotide variation map was carried out using the R package ggplot2 in the R v4.3.2 program. In addition, we performed synteny and rearrangement analyses for nine species using the Mauve tool (Darling et al. 2004) in Geneious Prime 2023.2.1 (Kearse et al. 2012).

Two types of repetitive sequences were identified. Simple repeat sequences (SSRs) were screened using MISA‐web (Beier et al. 2017), with minimum repeat lengths of 10, 5, 4, 3, 3, and 3 defining mono‐, di‐, tri‐, tetra‐, penta‐, and hexanucleotide repeat units, respectively. The distribution of SSRs in the plastomes was also counted. The identification of scattered repetitive sequences, including forward, reverse, complementary, and palindrome repeats, was performed on the REPuter (Kurtz et al. 2001) with the parameters (‐c ‐f ‐p ‐r ‐l 30‐best 5000).

### 2.4 Molecular Evolution Analysis

To determine codon usage patterns, we performed codon usage estimation for the nine species using CodonW v1.4.2 software (https://codonw.sourceforge.net/, accessed on February 26, 2024) (translation table = 11), and calculated the shared CDS sequences for each species' RSCU values. Subsequently, we visualized the RSCU values using TBTools v2.067 (Chen, Wu, et al. 2023) and clustered them by different species. The effective number of codons (ENC) is an important measure of the degree of preference for the biased use of synonymous codons, and it is well known that highly expressed genes also have a large degree of codon preference, which results in smaller ENC values (Xu et al. 2011). Therefore, in addition to the overall codon usage rate, we further listed other codon usage indices such as ENC and the distribution of GC content at codon positions. The GC content at the first, second, and third positions of codons (GC1, GC2, and GC3, respectively) was calculated using the CHIP and CUSP plugins in EMBOSS (Rice et al. 2000). We also conducted selection pressure analysis by calculating the Ka/Ks ratio (ω) for each gene using the shared CDSs with KaKs_Calculator v2.0 (Wang et al. 2010), selected the bacterial and plant plastid code, and used the YN method. In cases where Ka/Ks indicated “NA” due to Ks = 0 (no substitutions in alignment or 100% match), it was replaced with 0. An ω = 1, ω > 1, and ω < 1 indicate neutral, positive, and purifying selection, respectively.

### 2.5 Phylogenetic Analysis

To reconstruct the phylogenetic relationship, we integrated two data sets: (1) Newly generated CDS data extracted from 16 complete plastomes representing five Alismataceae genera (Wang et al. 2018) (*Alisma* [*n* = 7], *Luronium* [*n* = 1], *Caldesia* [*n* = 4], *Sagittaria* [*n* = 3], and *Aquarius* [*n* = 1]), supplemented with four additional outgroup plastomes; (2) published CDSs from 30 species, including four outgroup taxa, from Li et al. (2022). A total of eight plasmid sequences from *Stratiotes aloides* , *Ottelia acuminata*, *Butomus umbellatus* , *Blyxa japonica*, *Thalassia hemprichii* , *Enhalus acoroides* , *Butomus umbellatus* , and *Halophila beccarii* were used as outgroups. First, we extracted the coding regions from the plastomes of *A. macrophyllus* and its closely related species. Sixty‐one coding genes from 16 species were compiled into a 61,601 bp data matrix using Python in conjunction with the CDS data from Li et al. (2022) and were then aligned using MAFFT v7.475 (Katoh and Standley 2013). The best model (TVM + F + R3) was inferred based on the concatenated matrix. The phylogenetic tree was constructed using the maximum likelihood (ML) method implemented in IQ‐TREE v1.6.8 (Du et al. 2022; Nguyen et al. 2015) with 1000 bootstrap replicates. Additionally, the best Bayesian model (GTR + I + G) was inferred using jModelTest v2.1.9 based on the Bayesian Information Criterion (BIC) (Darriba et al. 2012; Xiang et al. 2024). The Bayesian phylogenetic tree was constructed using MrBayes 3.2.7a (Miller et al. 2010) on the CIPRES online platform (https://www.phylo.org/portal2/login!input.action). Tree visualization was performed in Figtree v1.4.4 (http://tree.bio.ed.ac.uk/software/figtree/).

## 3 Results

### 3.1 Characteristics of the *A. macrophyllus* Plastome

**FIGURE 1:**
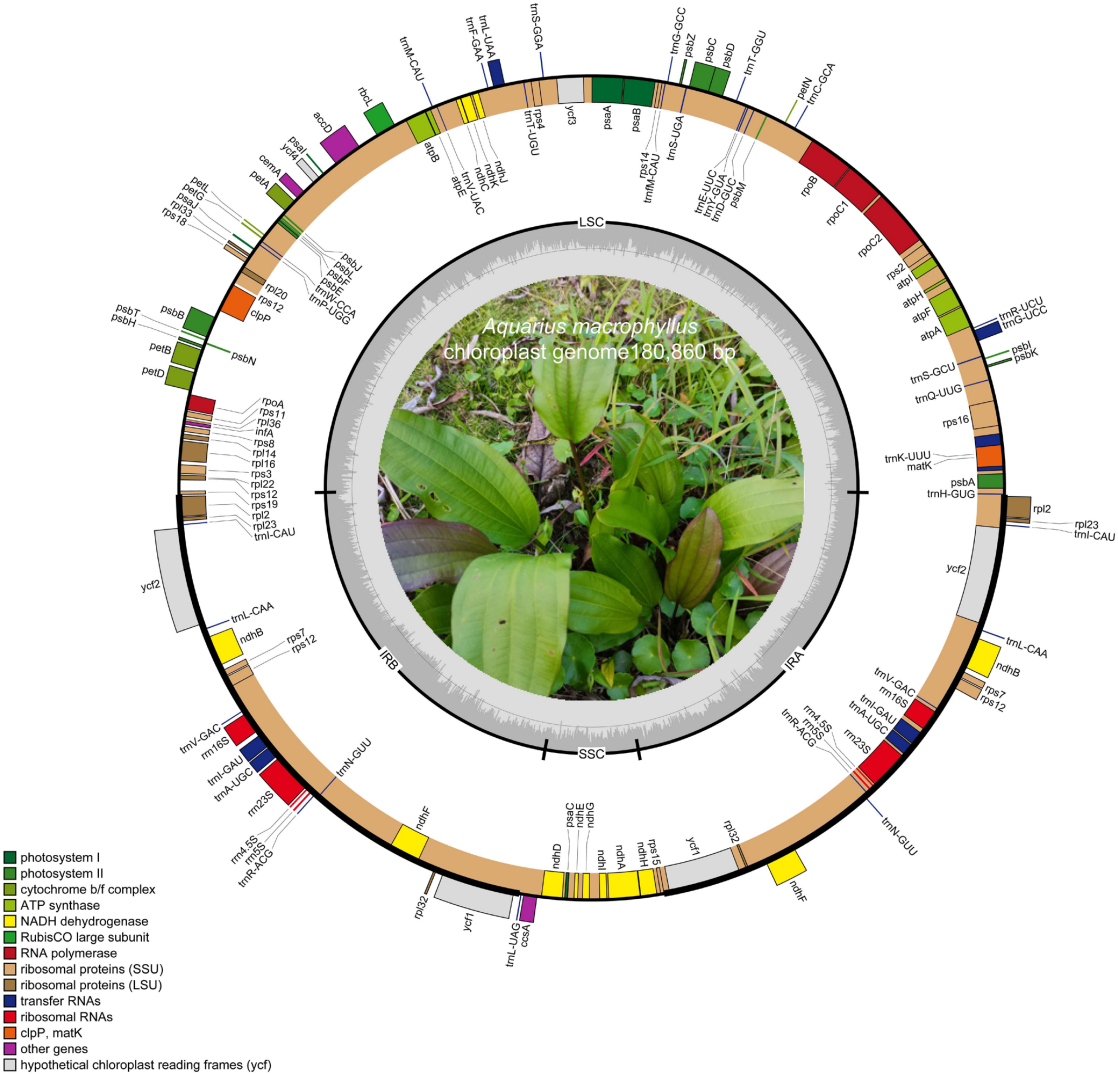
Structural map of the complete plastome of *Aquarius macrophyllus*. Genes inside the circle are transcribed clockwise, and genes outside the circle are transcribed counterclockwise. Genes were color‐coded according to their annotated function. The GC content of the plastome was indicated by the dashed area.

We sequenced the plastome of *A. macrophyllus* and confirmed the annotation accuracy using CPGView (Liu et al. 2023) and GB2Sequin (Lehwark and Greiner 2019). The complete plastome of *A. macrophyllus* was 180,860 bp in length, including two inverted repeat sequences (IRa and IRb) of 39,664 bp each, separated by an LSC region (91,401 bp) and an SSC region (10,131 bp) (Figure 1). The total GC content of the *A. macrophyllus* plastome was 38.10%, with GC content in the LSC, SSC, and IR regions being 36.73%, 33.57%, and 40.30%, respectively. A total of 133 genes were annotated, including 8 rRNA genes (Table S1), 37 tRNA genes, and 88 protein‐coding genes. Among these, the genes *ndhB*, *ndhF*, *rpl2*, *rpl23*, *rpl32*, *rps7*, *rrn16S*, *rrn23S*, *rrn4.5S*, *rrn5S*, *trnA‐UGC*, *trnI‐CAU*, *trnI‐GAU*, *trnL‐CAA*, *trnN‐GUU*, *trnR‐ACG*, *trnV‐GAC*, *ycf1*, and *ycf2* were duplicated in IR regions (Table S1). Among a total of 113 distinct genes, 14 contain one intron (9 protein‐coding genes and 5 tRNA genes) and two protein‐coding genes (*ycf3* and *clpP*) contained two introns (Figure S1, Table S2). The *rps12* gene was trans‐spliced (Figure S2).

### 3.2 Comparative Analysis

Comparative analysis of the plastomes of nine Alismataceae plants showed a length variation ranging from 159 to 180 kb (Table 1). *Luronium natans* exhibited the shortest plastomes, whereas *A. macrophyllus* possessed the longest. The IR length varied from 25 to 39 kb, with *Alisma* having the shortest IR, but its SSC region occupies the largest proportion of the entire plastome. *A. macrophyllus* possessed the largest IR (39,664 bp), and this IR region expands to include the *ndhF‐ycf1* region, which is typically located in the small single‐copy (SSC) region in other plastomes. This IR expansion resulted in the duplication of the *ndhF‐ycf1* region, contributing to the increased size of the *A. macrophyllus* plastome uniquely. *A. macrophyllus* had the highest GC content (38.10%) among all the nine species, while the remaining taxa displayed a narrower range of 36%–37% (Table 1). Moreover, the highest GC content was found in the IR region, followed by the LSC region, and the lowest in the SSC region (Table 1). There were 130–137 functional genes in the nine plastomes, of which 113–115 were unique, including 74–75 protein‐coding genes, 29–30 tRNA genes, and 4 rRNA genes. In *Caldesia parnassifolia*, two plastid genes (*rps19* and *ycf1*) were lost, whereas lineage‐specific gene duplications were identified in other taxa: *ndhH* (*Sagittaria* spp.), *ndhF* ( *A. macrophyllus* ), *ycf15* (*Sagittaria lichuanensis*, *S. trifolia* , *Caldesia grandis*, *C. parnassiflia*), and *ycf68* ( *S. lichuanensis* , *S. trifolia* ). Notably, the *ycf15* and *ycf68* duplications were exclusively restricted to these lineages, with this gene not found in other species examined (Table S3). In addition, loss of introns was detected. For instance, the *trnV‐UAC* gene of *A. macrophyllus* plastome lacked an intron compared to other species (Tables S1 and S2).

**TABLE 1:** Comparison of the plastome features of nine Alismataceae species.

Species	Genome size (bp)	LSC length (bp) (GC content)	SSC length (bp) (GC content)	IR length (bp) (GC content)	Gene	tRNA	rRNA	Protein‐coding gene	GC content (%)
*Aquarius macrophyllus*	180,860	91,401 (36.73%)	10,131 (33.57%)	39,664 (40.30%)	133	37	8	88	38.10
*Sagittaria graminea*	176,871	98,820 (34.94%)	12,875 (31.15%)	32,588 (41.27%)	132	37	8	87	37.00
*Sagittaria lichuanensis*	179,007	99,125 (34.70%)	13,278 (30.63%)	33,302 (41.25%)	137	37	8	88	36.80
*Sagittaria trifolia*	177,417	97,435 (34.82%)	12,580 (31.46%)	33,691 (41.22%)	137	37	8	88	37.00
*Caldesia grandis*	173,124	90,983 (35.19%)	13,643 (31.31%)	34,249 (39.77%)	132	37	8	87	36.70
*Caldesia parnassifolia*	173,051	95,796 (35.69%)	13,655 (31.46%)	31,800 (41.06%)	130	36	8	86	37.30
*Alisma plantago‐aquatica*	159,686	89,425 (33.70%)	19,753 (29.20%)	25,254 (42.80%)	130	37	8	85	36.00
*Alisma canaliculatum*	159,471	89,249 (33.70%)	19,498 (29.30%)	25,362 (42.7%)	130	37	8	85	36.00
*Luronium natans*	159,063	89,203 (33.70%)	19,612 (29.3%)	25,124 (42.9%)	130	37	8	85	36.10

**FIGURE 2:**
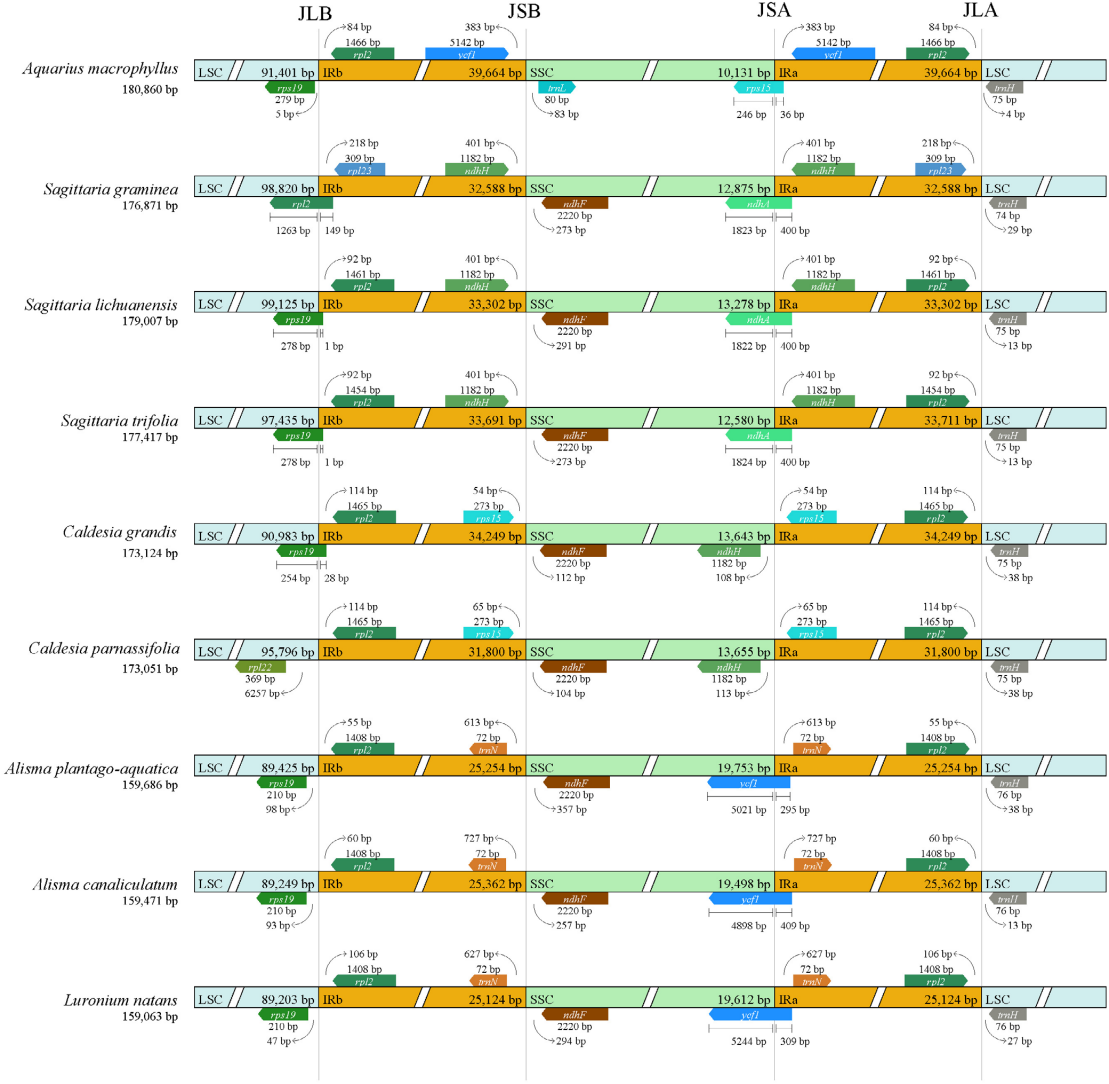
Comparison of plastome borders of LSC, SSC, and IRs among the nine species of Alismataceae. JLB, junction between LSC and IRb; JSB, junction between SSC and IRb; JSA, junction between SSC and IRa; JLA, junction between LSC and IRa.

The IR boundary contraction and expansion of Alismataceae species were displayed (Figure 2). For example, the LSC and IRb (JLB) junction started just after *rps19* (5*–*98 bp) in *A. macrophyllus* , *Alisma* spp., and *L. natans* . However, structural variations were observed in other taxa: (i) *S. graminea*, where the *rpl2* gene spanned the JLB boundary, and (ii) *S. lichuanensis* the *rps1*9 gene overlapping the JLB junction, a feature shared with *S. trifolia* and *C. grandis* (Figure 2). No genes were found to span the IRb/SSC junction (JSB) in any of the species examined. The *ndhF* gene was consistently located downstream of the JSB (104–357 bp), with the exception of *A. macrophyllus*. In *Sagittaria* spp., the *ndhH* gene was located within the IRb region, specifically 401 bp proximal to the junction (Figure 2). The IR expansion resulted in the relocation of *ndhH* to the inverted repeat (IR) regions, a structural reorganization not observed in other Alismataceae genera (e.g., *Caldesia*, *Aquarius*, *Alisma*, *Luronium*), where *ndhH* remains within the small single‐copy (SSC) region (Figure 2). Similarly, *rps15* in *Caldesia* spp. was localized in the IRb region, positioned 54–65 bp from the junction. However, in the *A. macrophyllus* plastome, the *ndhF* and *rpl32* genes were confined within the IRb due to the expansion of the IR region, while the *ycf1* gene was adjacent to the JSB, 383 bp from the junction. In *S. graminea*, *S. lichuanensis* , and *S. trifolia* , the *ndhA* genes were located at the SSC/IRa junction (JSA) and extended 1823, 1822, and 1824 bp into the SSC region, respectively, whereas this boundary position is occupied by *ycf1* in *Alisma* spp. and *L. natans* . The boundary genes of *C. grandis* and *C. parnassifolia* were *ndhH* (SSC) and *rps15* (IR), 108, 113, and 54, 65 bp distant from the junction, respectively. Especially, the extension of the IR region in *A. macrophyllus* caused *rps15* to be located in the JSA and extend 246 bp to the SSC region. The IRa/LSC junction (JLA) was between the *rpl2* and *trnH* genes in all species except for *S. graminea* , where the IRa boundary was *rpl23*. The *trnH* gene was situated in the LSC region of nine species, 4–38 bp away from the boundary point.

**FIGURE 3:**
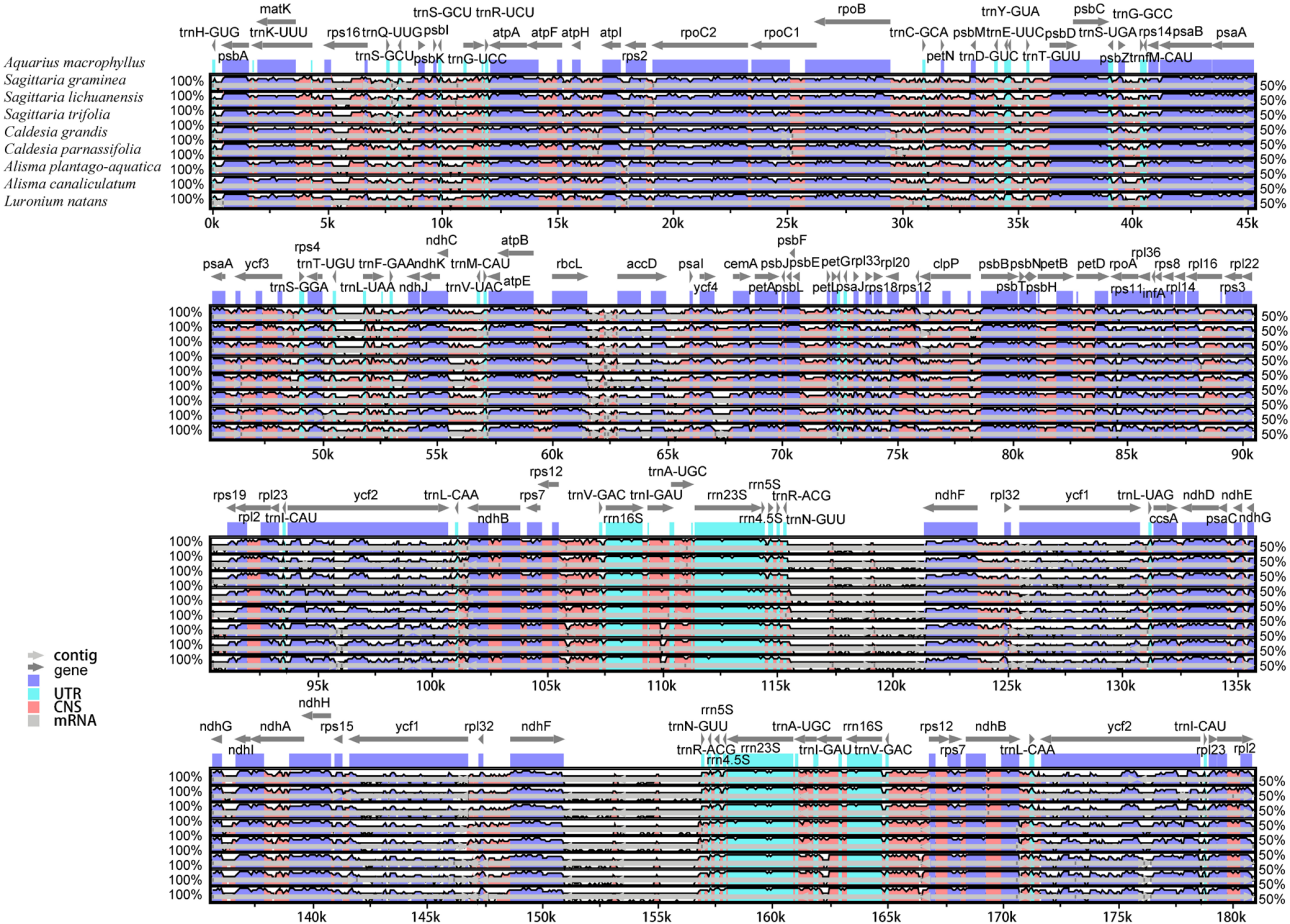
Sequence identity plots (mVISTA) among nine plastomes of Alismataceae, using *A. macrophyllus* as a reference genome. Arrows indicate the annotated genes and their transcriptional direction. Genome regions are color coded as exon, untranslated region (UTR), conserved noncoding sequences (CNS), and mRNA.

The mVISTA results showed that the four parts of the plastomes were arranged in a relatively consistent order, but significant variations in the noncoding regions of the genes existed (Figure 3). Compared with *A. macrophyllus* , two sequence inversions were detected by synteny and rearrangement analyses. One inversion of 5.6 kb was from the gene *trnS‐GCU* to *trnQ‐UUG* (*trnS‐GCU* + *psbI* + *psbK* + *trnQ‐UUG*) in *Sagittaria*, and another inversion of 6.4 kb was from *trnV‐UAC* to *rbcL* (*trnV‐UAC* + *trnM‐CAU* + *atpE* + *atpB* + *rbcL*) in *Caldesia* (Figure S3). In addition, combined with the SSR analysis, we found that SSRs were rich at the inversion in *Sagittaria* at IGS (*rps16*, *trnS‐GCU*), IGS (*psbK*, *trnQ‐UUG*), and *trnG‐UCC* intron1. Similarly, SSRs were found to be rich at the inversion terminal ends in *Caldesia* at IGS (*trnV‐UAC*, *accD*) and IGS (*accD*‐*psaI*) (Table S4).

**FIGURE 4:**
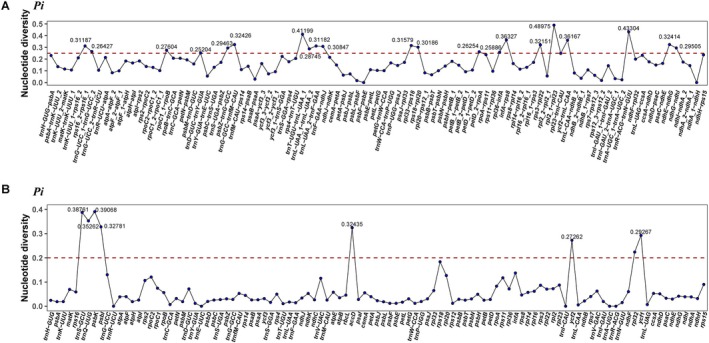
The Pi values of the nine plastomes. (A) Pi values of IGS. *Notes:* _1 indicates the start of the first exon of the gene. (B) Pi values of protein‐coding genes.

All the shared genes and intergenic regions (IGS) were extracted to calculate nucleotide diversity (Pi) (Figure 4, Table S5). The results show that the IGS region (average Pi = 0.173) had a higher polymorphism than the gene region (average Pi = 0.064). The most variable gene regions included: *rpl32*, *ycf1*, *ycf2*, *accD*, *psbI*, *psbK*, *trnQ‐UUG*, and *trnS‐GCU* (Pi > 0.2), five of which were located in the LSC region and three in the IR region. The IGS regions with large variation include *trnD‐GUC‐trnY‐GUA*, *rpl36‐infA*, *petD_2‐rpoA*, *rpoC1_1‐rpoB*, *psbZ‐trnG‐GCC*, *ndhG‐ndhI*, *rps18‐rpl20*, *trnF‐GAA‐ndhJ*, *trnL‐UAA_2‐trnF‐GAA*, *rpl33‐rps18*, *rps3‐rpl22*, *ndhE‐ndhG*, *trnG‐GCC‐trnfM‐CAU*, *ycf2‐trnL‐CAA*, *infA‐rps8*, *trnT‐UGU‐trnL‐UAA_1*and *trnR‐ACG‐trnN‐GUU*, *rpl2_1‐rpl23*, *trnG‐UCC* intron, *trnL‐UAA* intron, and *rps16* intron (Pi > 0.25). Of these, 16 were located in the LSC region, two in the SSC region, and three in the IR region.

### 3.3 Repeat Sequence Analysis

**FIGURE 5:**
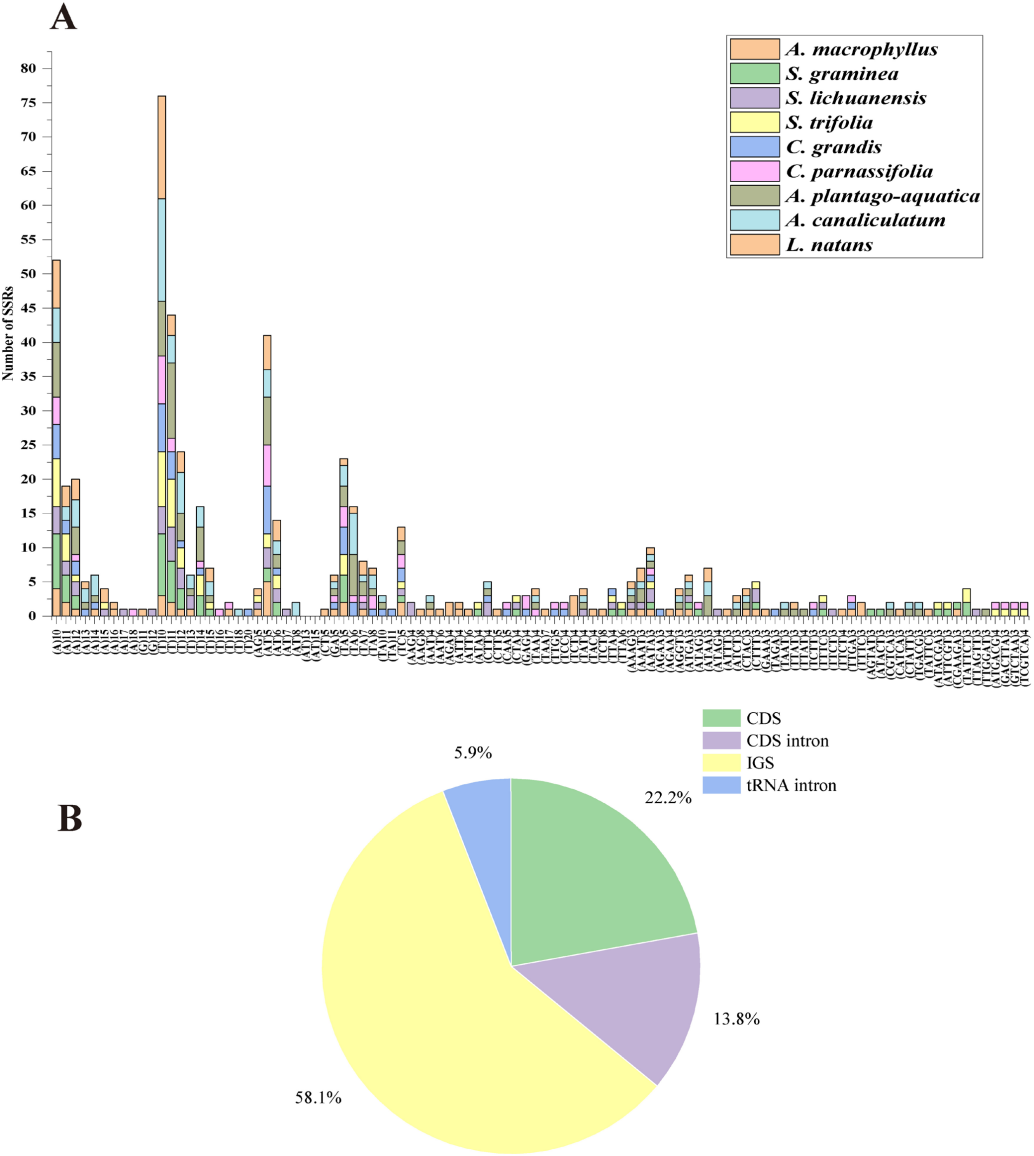
Simple sequence repeats (SSRs) in the nine plastomes. (A) Number of SSRs by length. (B) Distribution of SSR loci. CDS, coding DNA sequence; IGS, intergenic spacer region.

Within the nine plastomes, MISA detected a total of 532 SSRs. Among these, *A. canaliculatum* and *A. plantago‐aquatica* exhibited the highest SSR counts (73/66), whereas *A. macrophyllus* and *C. parnassifolia* had the lowest (48/49) (Table S6). *Sagittaria graminea*, *S. lichuanensis* , *S. trifolia* , and *L. natans* possessed 61, 55, 57, and 63 SSRs, respectively. Among the SSRs analyzed, a total of 266 mononucleotide SSRs were identified, accounting for 50% of the SSRs, and 121 dinucleotides with two patterns (AG/CT and AT/AT), which represented 23%. Additionally, there were five trinucleotide repeat types, accounting for 8% of the SSRs, and 73 tetranucleotides with diverse patterns (AAAG/CTTT, AAAT/ATTT, AATC/ATTG, AATG/ATTC, AATT/AATT [only *Alisma* spp.], AGAT/ATCT, and ACCT/AGGT), which represented 14% of the SSRs. Only 10 pentanucleotide repeats were present in *S. graminea* , *Alisma* spp., and *L. natans* , accounting for 2% of the SSRs. Finally, there were 19 hexanucleotides with 13 repeat types, accounting for 3%. The dinucleotide repeat form was present in all nine species, but only *A. macrophyllus* , *Alisma* spp., and *L. natans* had repeats in the form of ACCT/AGGT (Figure 5A; Table S6). The analysis revealed that most of the SSR motifs were located in the IGS (56.4%), followed by CDS (22.6%) and the CDS intron (14.3%) (Figure 5B), which may be due to the high mutation rate in the intergenic spacer region (IGS).

**FIGURE 6:**
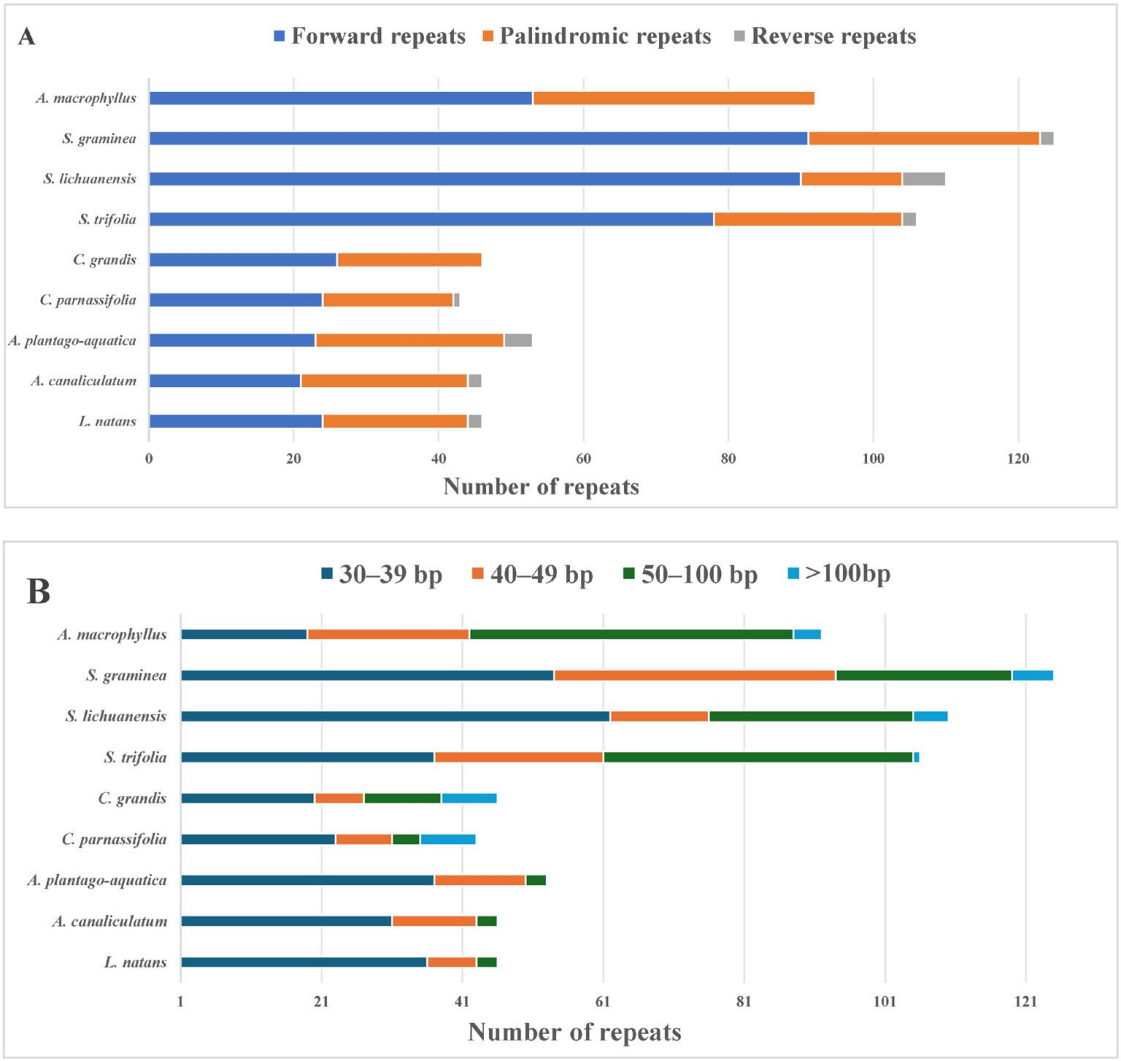
Repeat sequences in nine plastomes of Alismataceae. (A) Frequency of repeat types. (B) Frequency of repeats by length.

REPuter analysis showed that the nine plastomes presented three types of scattered repetitive sequences, including forward (F), reverse (R), and palindromic (P), and no complementary (C) repetitive sequences were detected (Table S7). A total of 667 repeat sequences, including 430 F, 218 P, and 19 R repeats, were detected. F and P repeats were the most common types (Figure 6A). *S. graminea* had the highest number of repeats (125), whereas *C. parnassifolia* had the lowest (Wu, Li, et al. 2024). The distribution of repeat lengths indicated that 48% of repeats were 30–39 bp long, whereas 25% ranged from 50 to 100 bp, and 5% exceeded 100 bp (Figure 6B).

### 3.4 Codon Usage Bias Analysis

Relative synonymous codon usage (RSCU) indicates the ratio of actual to expected usage frequencies of codons. The results showed that the RSCU values of all species were very similar (Table S8). The total number of codons varied, with *A. macrophyllus* having the highest (60,286) and *L. natans* the lowest (53,021) (Figure S4, Table S8). Among the 61 codons in the plastomes of the nine Alismataceae plants, except for AUG encoding methionine (Met) and UGG encoding tryptophan (Trp) (RSCU = 1), 28 codons had RSCU values > 1 and mainly ended with A and U, which were high‐frequency codons. Among them, the AGA codon corresponding to arginine (Arg) had the highest recognition rate, with an average RSCU of 2.016. The remaining 31 codons showed low preference, with RSCU values < 1 (Table S8). In addition, the average effective codon (ENC) for all plants was 54.65, and the average GC content of the third base of synonymous codons ranged from35.41% ( *A. canaliculatum* ) to 38.44% ( *A. macrophyllus* ), suggesting that codon bias is weak in Alismataceae species, with a greater predominance of A/U ending codons.

### 3.5 Selection Pressure Analysis

**FIGURE 7:**
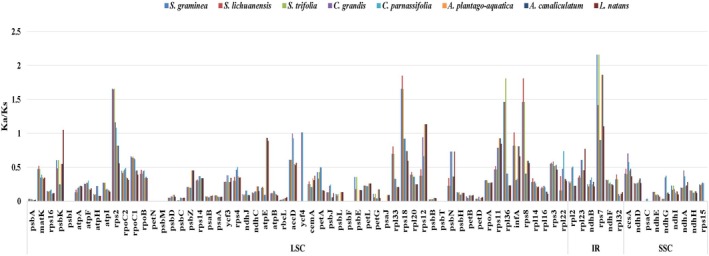
The Ka/Ks ratio of 73 protein‐coding genes shared in nine plastomes with *A. macrophyllus* as a reference.

Using *A. macrophyllus* as the reference, we calculated synonymous (Ks) and nonsynonymous (Ka) substitution rates through pairwise comparisons with each species of *Caldesia*, *Sagittaria*, *Alisma*, and *Luronium*. The analysis showed that Ka/Ks ratios did not differ significantly across regions (IR, SSC, and LSC), but gene‐specific and intergeneric specificity were detected (Figure 7). The mean Ka/Ks value of the 73 protein‐coding genes was 0.30. The most conserved genes were *psbI*, *petN*, *psbM*, *psbF*, *psbT*, and *psaC*, with mean Ka/Ks values ranging from 0 to 0.01, which indicated a high purification selection pressure. Chloroplast‐encoded ribosomal protein genes (e.g., *rps2*, *rps18*, *rps7*) exhibited Ka/Ks > 1, suggesting positive selection acting on these loci, potentially linked to the adaptive evolution of the translation machinery. Furthermore, these positively selected genes showed intergeneric specificity, with *Sagittaria* species having significantly higher Ka/Ks values than *Caldesia* species. Compared with *A. macrophyllus* , *Caldesia* species showed higher Ka/Ks values in the *ycf4* gene (Table S9). The Ka/Ks values of the genes *rpoC1*, *accD*, *rpl36*, *rps12*, *rps11*, *infA*, *rps3*, *psbK*, and *rps8* were in the range of 0.5–1, implying a relaxed selection pressure (Figure 7). Seventy‐six percent of genes had Ka/Ks values between 0.02 and 0.49 (Table S9), suggesting that the majority of genes in the Alismataceae plastomes are under purifying selection.

### 3.6 Phylogenetic Analysis

**FIGURE 8:**
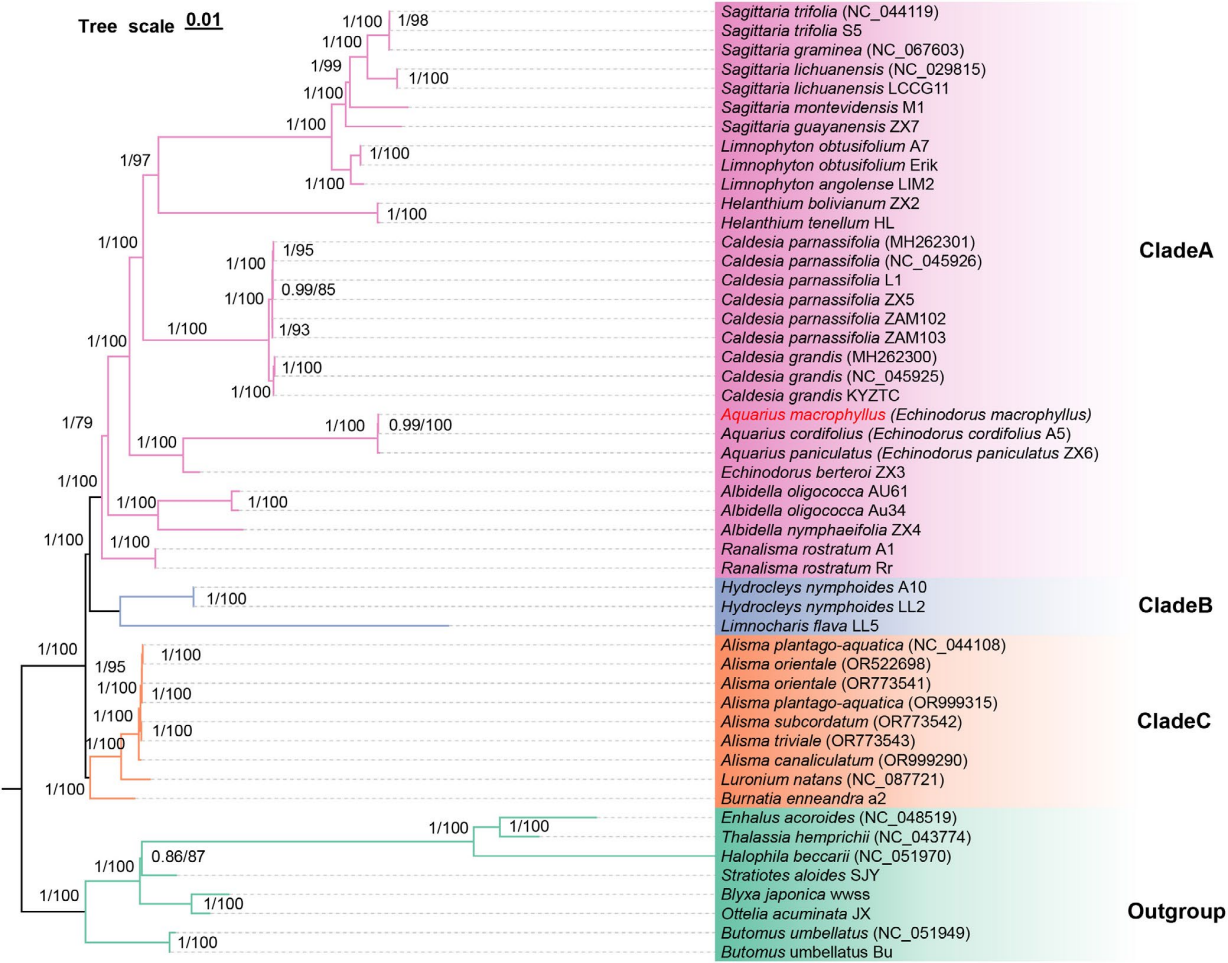
Phylogeny of Alismataceae based on 61 plastome protein‐coding genes from 50 species.

To analyze the phylogenetic relationships of *A. macrophyllus* in Alismataceae, a phylogenetic tree was reconstructed using 61 protein‐coding regions shared in the plastomes of 50 species (Table S10). Maximum likelihood (ML) and Bayesian inference (BI) methods yielded identical topologies (Figure 8). Three clades (A, B, C) were resolved. Clade A included eight generasuch as *Albidella*, *Aquarius*, *Caldesia*, *Echinodorus*, *Helanthium*, *Limnophyton*, *Ranalisma*, *and Sagittaria*. Clade B was composed of *Hydrocleys* and *Limnocharis*. It was observed that the two species *A. plantago‐aquatica* and *A. orientale* in Clade C are widely distributed in Eurasia, and they cannot be clearly delineated. All the current genera were well supported as monophylies, with *Alisma*, *Luronium*, and *Burnatia* at the basal position. Phylogenetic results showed that three species of *Aquarius* formed a monophyletic group (ML/BS = 100%, BI/PP = 1.0), being sister to *Echinodorus* with strong support. *A. macrophyllus* showed a close relationship with *A. cordifolius* in the *Aquarius* clade (Figure 8). The clade of *Echinodorus* + *Aquarius* was sister to *Sagittaria* + *Limnophyton* + *Helanthium* + *Caldesia* (BS = 100, PP = 1.0), followed by *Albidella* + *Ranalisma*.

## 4 Discussion

Since most of Alismataceae species are aquatic plants, it is crucial to study the phylogeny and plastome evolution due to their morphological degeneration and presumed convergent adaptation to aquatic habitats (The Angiosperm Phylogeny Group 2016; Lehtonen and Myllys 2008; Les et al. 1991). However, to date, no complete plastomes of *Aquarius* species were available, although the plastome of *Aquarius grisebachii* incorrectly assembled with part of the IR region missing has been published in NCBI (OR999317). For the other two species of *A. cordifolius* and *A. paniculatus* , only CDS data were available (Chen et al. 2012). Therefore, in the present study, we sequenced and assembled the first complete plastome of *A. macrophyllus* and performed a comparative analysis with four genera of Alismataceae. The plastome of *A. macrophyllus* exhibits a conventional quadripartite structure consistent with most angiosperms (Zhou et al. 2022; Weng et al. 2023; Zhu et al. 2024). The total length of the *A. macrophyllus* plastome is 180,860 bp, which is a notably large genome size among Alismataceae species. The GC content, protein‐coding genes, rRNAs, and tRNAs of the plastome of *A. macrophyllus* were similar to those of other Alismataceae species (Liu et al. 2009; Liang et al. 2019), indicating the conservation of the plastome sequences.

In general, angiosperm plastomes are highly conserved compared to nuclear sequence (Drouin et al. 2008). The complete plastid genomes of *A. macrophyllus* and *Sagittaria* are notably larger than those of *Alisma* and *Caldesia* within the Alismataceae family. Furthermore, the plastomes of the nine species exhibit variations, including intron loss, gene duplication, and gene loss. Gene loss was frequently observed in flowering plants (Jansen et al. 2007). Multiple intron losses were discovered in the genes of *accD*, *clpP*, and *rpoC1* in the Poales (Wu, Li, et al. 2024). The *trnV*‐*UAC* gene lacks an intron in *A. macrophyllus* but is present in the other eight plastomes, suggesting intron loss during species divergence. Whether the *trnV*‐*UAC* intron loss is specific to *Aquarius* requires more plastomes data of *Aquarius* to verify. The gene loss of *rps19* was unique in *C. parnassifolia* compared with the other eight plastomes. In aquatic species of Alismatidae, three independent *ndh* losses was revealed, which might explain the mechanism of reducing photooxidative stresses in submerged habitats (Li, Lehtonen, et al. 2023). Gene duplication is mainly due to IR expansion, such as those found in *Eleocharis* (Cyperaceae) (Lee et al. 2020). In the present study, lineage‐specific gene duplication events were observed: *ndhH* exhibited two copies exclusively in *Sagittaria* species, while the *ndhF* duplication was uniquely retained in *A. macrophyllus* . In contrast, *Alisma*, *Caldesia*, and *Luronium* species retained only single copies of both genes. This copy number variation, primarily caused by IR expansion that relocated these genes to the IR regions, may reflect divergent genomic strategies for adaptation to aquatic niches, with duplicated *ndh* genes potentially enhancing photosynthetic flexibility in emergent lifeforms (Silva et al. 2016). These similar features of plastome genomic variation have been commonly reported in previous studies, such as the gene duplication of *trnG‐GCC* in Costaceae species (Li et al. 2024), and the loss of the *psbE*, *trnG‐GCC* genes in *Amorphophallus* (Araceae) (Liu et al. 2019). While most *ycf* genes remain functionally unannotated in plastomes, experimental studies have established that *ycf1* and *ycf2* encode indispensable proteins for cellular survival (Drescher et al. 2000). A pronounced variation was observed among the genera of Alismataceae with respect to the type, number, and length of *ycf* genes. For instance, *C. parnassifolia* was found to be devoid of the *ycf1* gene, while *Sagittaria* spp. and *A. macrophyllus* exhibited two copies of this gene. In contrast, *Alisma* and *Luronium* sequences contained only a single *ycf1* gene (Lan et al. 2024). Moreover, *ycf15* and *ycf68* genes were exclusively observed in *S. lichuanensis* and *S. trifolia* , with these two genes being absent in the remaining nine species.

IR contraction and expansion are important evolutionary events in plant plastomes, which are closely associated with genome size variation and gene duplication (Wanichthanarak et al. 2023; Wang et al. 2022). For example, the gene composition on both sides of the IR‐LSC junctions can be used to distinguish monocots from other angiosperms, as the former has a *trnH‐rps19* gene cluster near these junctions (Luo et al. 2016). A similar feature was observed in our study. In addition, the *ndhF* gene in *A. macrophyllus* was lost at the IRb/SSC junction (Figure 2), which may be related to the expansion of its IR region, resulting in genetic differences adjacent to the IR region boundary (Li et al. 2024). Currently, complete plastome sequences are only available for *Aquarius macrophyllus*. The other two *Aquarius* species only have CDS sequences, not complete plastome sequences, and there is no way to know if *ndhF* is also duplicated. This difference highlights the unique structural variation in *A. macrophyllus* and suggests that IR expansion may play a role in gene duplication events in this species, but more complete chloroplast data are needed to validate *Aquarius*. The inversion from *rbcL* to *trnV‐UAC* was found to characterize a monophyly of six families in Alismatidae and independently occurred in *Caldesia grandis* as well (Li, Lehtonen, et al. 2023). Two inversions in *Sagittaria* and *Caldesia* species were also observed among the nine species. Genetic recombination events may be responsible for the generation of gene inversions, which are associated with the presence of short repeats at the end of inversions (Zhang and Chen 2022). The inversions identified in *Sagittaria* (5.6 kb spanning *trnS‐GCU* to *trnQ‐UUG*) and *Caldesia* (6.4 kb spanning *trnV‐UAC* to *rbcL*) align with patterns observed in Poales, where large inversions (> 1 kb) predominantly localize to the LSC region. These structural rearrangements may arise from replication slippage or homologous recombination mediated by flanking repeats or SSRs, as evidenced by the enrichment of SSRs at inversion termini (e.g., *rps16‐trnS‐GCU*) IGS in *Sagittaria* and (*trnV‐UAC‐accD*) IGS in *Caldesia*). Inversion in plastomes has been suggested to play a role in the adaptive evolution in plants (Mwanzia et al. 2019; Cui et al. 2006). For example, the Alismatidae plastome inversion was revealed to be linked to ancient polyploidization during the K–Pg boundary (66–74 Ma), where genomic instability potentially drove chloroplast restructuring under extreme environmental pressures (Li, Lehtonen, et al. 2023).

Recombination mediated by repetitive sequences increases the structural complexity of plastomes, providing valuable insights into genome structural variation and phylogenetics (Cao et al. 2024). Among the nine Alismataceae species, *A. macrophyllus* contains the fewest SSRs (Drescher et al. 2000). Most SSRs are located in intergenic regions and predominantly consist of A/T bases, consistent with trends observed in other higher plants (Zhang and Chen 2022; Hong et al. 2020; Ebrahimi‐Fallah and Askari 2024). SSRs are important molecular markers for the study of plant population genetics, evolution and ecology (Xu et al. 2018). The SSRs we detected in the nine plastomes posed potential value as molecular markers for future population genetics studies at the genus or species level. Repeat sequences play an important role in sequence recombination and plastid variation (Silva et al. 2019). We detected the presence of 25% long scattered repeats (50–100 bp), with long repeats contributing significantly to plastome rearrangement and stabilization (Shi, Shi, et al. 2023; Shi et al. 2024). The presence of palindromic repeats (reverse complements) can form hairpin structures, which may function in replication mechanisms (Kurtz et al. 2001).

Codon usage preferences have important implications for plant evolution and can be used to study evolutionary processes at the molecular level (Geng et al. 2022). To better understand the evolution of *A. macrophyllus* and its closely related species, it is crucial to study codon preferences that influence plastome evolution (Guo et al. 2022). RSCU data reveal a preference for amino acids with A/U‐ending codons in Alismataceae species. The AGA codon (Arg) demonstrated the most pronounced codon usage bias (average RSCU = 2.016), a phenomenon that has also been observed in eight Sapindaceae species. This observation suggests a conserved preference for this codon across divergent angiosperm lineages. The weak overall codon bias (average ENC = 54.65) and low GC content at the third codon position (35.41%–38.44%) align with the typical AT‐rich nature of chloroplast genomes, where mutational bias and relaxed selection pressure may jointly shape synonymous codon usage (Song et al. 2024). This result supports the influence of codon preferences on the diversification of species (Shahzadi et al. 2020).

Highly variable regions in plastomes are useful molecular markers to distinguish between closely related species (Chen, Zhang, et al. 2022). Through Pi analysis, we identified highly variable regions (*rpl32*, *ycf1*, *ycf2*, *accD*, *psbI*, *psbK*, *trnQ‐UUG*, and *trnS‐GCU*) for *A. macrophyllus* and related species in Alismataceae. It is noteworthy that several genes exhibited marked variability (Pi > 0.2) and also demonstrated inversion, including *psbI*, *psbK*, *trnQ‐UUG*, and *trnS‐GCU*. The majority of highly variable genes and spacer regions are found in large single copy (LSC) regions. These regions are indicative of diversity and are therefore favorable tools for population genetics and species identification across genera in Alismataceae. For instance, *trnE‐UUC‐trnT‐GGU*, *accD*, *clpP*, and *ycf2* have been reported as DNA barcodes to distinguish related species (Wang, Huo, et al. 2024).

The Ka/Ks ratio is commonly used to estimate the selective pressures and evolutionary rates of protein‐coding genes (Ivanova et al. 2017). Different mechanisms, such as selective pressure, mutational load, and localized hypermutation, have been found to shape substitution rates in plant organelle genomes (Wang, Kan, et al. 2024). For most plants, plastid mutation rates vary within a relatively narrow range. However, exceptional rate disparities have been reported in many comparisons of organelle genomes, such as in *Selaginella* (Selaginellaceae) (Xu et al. 2018) and *Physalis* (Solanaceae) (Zhang et al. 2023). The predominance of purifying selection (Ka/Ks < 1.96%) in most CDS of the nine plastomes in this study indicates their conservation. Compared to *A. macrophyllus* , genes with Ka/Ks ratios > 1 indicated positive selection, including *rps2*, *rps18*, and *rps7*, which encode ribosomal proteins. Their adaptive evolution was associated with protein synthesis in angiosperm plastids (Wu et al. 2018). These positively selected genes also showed intergeneric specificity that *Sagittaria* species have higher Ka/Ks values than *Caldesia* species, implying genome‐specific selective pressure across genera in Alismataceae. Furthermore, *Alisma* spp. and *L. natans* displayed considerably elevated Ka/Ks values in the ribosomal protein genes *rps7* and *rps12*. These findings signify divergent evolutionary pressures on the translational apparatus across Alismataceae species. The elevated Ka/Ks ratio of *ycf4* in *Caldesia* species compared to *Sagittaria* suggests that certain genes may experience differential selection pressures across genera. The functional roles of these genes, particularly in self‐replication and other plastid processes, suggest they may contribute to lineage‐specific adaptations in Alismataceae. While some studies have linked plastid gene selection to aquatic adaptation, further functional validation is needed to confirm the evolutionary significance of these patterns (Chen, Zang, et al. 2023).

Previous studies based on nuclear ITS and ptDNA (*psbA*, *rbcL*, *matK*, *rpoB*, and *rpoC1*) have clarified the phylogenetic framework of the family Alismataceae (The Angiosperm Phylogeny Group 2016; Ito and Tanaka 2023). However, some analyses contained low‐resolution branches due to limited nucleotide polymorphism sites and did not include *A. macrophyllus* (Chen et al. 2012; Guan et al. 2024). Plastomes, inherited through the maternal line, have been extensively employed for reconstructing phylogenetic relationships in recent years, replacing plastid fragments (Du et al. 2022). Li et al. (2022) constructed the first complete genus‐level plastid phylogeny of Alismataceae by using 78 genes and divide the Alismataceae into three major clades. The phylogenetic tree we reconstructed based on 61 shared plastome CDSs was mostly consistent with previous studies (Chen et al. 2012; The Angiosperm Phylogeny Group 2016; Guan et al. 2024). Three main clades were clustered comparatively. Lehtonen and Myllys (2008) separated *Helanthium* and the monotypic genus *Albidella* from *Echinodorus*, establishing a monophyletic genus *Echinodorus*. Our study supports the monophyly of *Aquarius*. Previous studies based on morphological data and plastid molecular markers suggested *Helanthium* as a subgenus of *Echinodorus* (Lehtonen 2006; Ross et al. 2016). However, Li (2022) and Lehtonen (2017) refuted the subordination between *Helanthium* and *Echinodorus*, and confirmed *Helanthium* as an independent clade sister to *Sagittaria* and *Limnophyton* (Li et al. 2022; Lehtonen 2017). For *Alisma orientale*, which is nested within the widespread *A. plantago‐aquatica* , additional nuclear data may be necessary in the future for the purpose of validation (Lan et al. 2024). Despite convergent vegetative traits between *A. orientale* and *A. plantago‐aquatica* , domestication‐driven divergence has likely shaped two distinct populations within the “ *A. plantago‐aquatica* complex,” differentiated by floral morphology (e.g., petal size, style curvature, stigma area) (Lan et al. 2024; Stant 1964). According to the systematic classification of angiosperms in the World Flora Online (https://wfoplantlist.org/, February 10, 2024), 26 species of *Echinodorus* have been placed in the new genus *Aquarius* (Govaerts et al. 2021; Plants of the World Online (POWO) 2023). Our phylogenetic analyses supported this classification and revealed its sister relationship with *Echinodorus*, consistent with previous studies separating *Echinodorus berteroi* as an independent branch (Chen et al. 2012; Lehtonen 2006; Lehtonen and Myllys 2008).

## 5 Conclusions

Our study reported the first complete plastome of *Aquarius macrophyllus*. Comparative analysis of *A. macrophyllus* and species of *Sagittaria*, *Caldesia*, *Alisma*, and *Luronium* revealed that the plastomes of these species are conserved globally, but there are also great sequence variations between the genera, including the IR expansion in *A. macrophyllus* , intron loss in *trnV‐UAC*, and lineage‐specific gene copy number variation. With *Aquarius* as a reference, two sequence inversions of 5.6 kb and −6.4 kb occurred in *Sagittaria* and *Caldesia*, respectively, which may be due to the rich SSRs at the end of the inversion. The *rps2*, *rps18*, and *rps7* genes were positively selected during evolution. Nine plastomes showed a preference for amino acids with A/U end codons. SSRs, dispersed repeat sequences, and variable regions have been identified with potential applications as molecular markers for future population genetics and phylogenetic studies. The phylogenetic relationships revealed the monophyly of *Aquarius* and its sister relationship with *Echinodorus* and support the establishment of genus *Aquarius*. Our study demonstrated the variation between *Aquarius* and related genera, and our data will provide more genetic information for future phylogenetic studies of Alismataceae.

## Author Contributions

**Jie Li:** data curation (equal), methodology (equal), software (equal), writing – original draft (equal). **Shengnan Wei:** formal analysis (equal), methodology (equal), software (equal), writing – original draft (equal). **Jianan Ying:** data curation (equal), resources (equal). **Qixiang Lu:** conceptualization (equal), funding acquisition (equal), supervision (equal), writing – review and editing (equal).

## Conflicts of Interest

The authors declare no conflicts of interest.

## Supporting information


**Figure S1.** Exon and intron structure mapping of cis‐spliced genes.

**Figure S2.** Detailed structure of the trans‐spliced gene rps12.

**Figure S3.** Synteny and rearrangements detected in nine plastomes. Color bars indicate syntenic blocks and connecting lines indicate corresponding blocks.

**Figure S4.** RSCU values heatmap of the codons in the nine Alismataceae plastomes.

**Table S1.** Coding genes of the *Aquarius macrophyllus* plastome.

**Table S2.** The genes with introns in the nine plastomes of Alismataceae.

**Table S3.** The genes with two copies in nine plastomes of Alismataceae.

**Table S4.** SSRs location in nine plastomes of Alismataceae.

**Table S5.** The Pi values of the chloroplast genomes among nine species.

**Table S6.** MISA analysis for the nine Alismataceae plastomes.

**Table S7.** Repeats numbers in nine Alismataceae plastomes.

**Table S8.** Summary of average relative synonymous codon usage (RSCU) of the codon usage in the Alismataceae cp genomes.

**Table S9.** Selection pressure data for nine plastomes.

**Table S10.** Species and 61 CDSs used for phylogenetics analysis.

## Data Availability

The plastome sequences of this study are openly available at the NCBI database (https://www.ncbi.nlm.nih.gov/nuccore/PP503046, GenBank accession number: PP503046).

## References

[ece371568-bib-0001] Beier, S. , T. Thiel , T. Münch , U. Scholz , and M. Mascher . 2017. “MISA‐Web: A Web Server for Microsatellite Prediction.” Bioinformatics 33: 2583–2585. 28398459 10.1093/bioinformatics/btx198 PMC5870701

[ece371568-bib-0002] Cao, Z. Y. , Y. Y. Qu , Y. Song , and P. Y. Xin . 2024. “Comparative Genomics and Phylogenetic Analysis of Chloroplast Genomes of Asian *Caryodaphnopsis* Taxa (Lauraceae).” Gene 907: 148259. 38346458 10.1016/j.gene.2024.148259

[ece371568-bib-0003] Chen, C. , Y. Wu , J. Li , et al. 2023. “TBtools‐II: A “One for all, all for One” Bioinformatics Platform for Biological Big‐Data Mining.” Molecular Plant 16: 1733–1742. 37740491 10.1016/j.molp.2023.09.010

[ece371568-bib-0004] Chen, J. , Y. Zang , S. Shang , et al. 2023. “Chloroplast Genomic Comparison Provides Insights Into the Evolution of Seagrasses.” BMC Plant Biology 23: 104. 36814193 10.1186/s12870-023-04119-9 PMC9945681

[ece371568-bib-0005] Chen, L. Y. , J. M. Chen , R. W. Gituru , T. D. Temam , and Q. F. Wang . 2012. “Generic Phylogeny and Historical Biogeography of Alismataceae, Inferred From Multiple DNA Sequences.” Molecular Phylogenetics and Evolution 63: 407–416. 22327014 10.1016/j.ympev.2012.01.016

[ece371568-bib-0006] Chen, M. M. , R. H. Wang , H. K. Sha , M. Z. Liu , J. Q. Tong , and Q. L. He . 2022. “The Complete Chloroplast Genome Sequence of *Spiraea × Vanhouttei* (Briot) Zabel (Rosaceae).” Mitochondrial DNA Part B Resources 7: 505–506. 35342798 10.1080/23802359.2022.2052369 PMC8942552

[ece371568-bib-0007] Chen, M. M. , M. Zhang , Z. S. Liang , and Q. L. He . 2022. “Characterization and Comparative Analysis of Chloroplast Genomes in Five *Uncaria* Species Endemic to China.” International Journal of Molecular Sciences 23: 11617. 36232915 10.3390/ijms231911617 PMC9569570

[ece371568-bib-0008] Cui, L. , J. Leebens‐Mack , L. S. Wang , et al. 2006. “Adaptive Evolution of Chloroplast Genome Structure Inferred Using a Parametric Bootstrap Approach.” BMC Evolutionary Biology 6: 13. 16469102 10.1186/1471-2148-6-13 PMC1421436

[ece371568-bib-0009] Darling, A. C. , B. Mau , F. R. Blattner , and N. T. Perna . 2004. “Mauve: Multiple Alignment of Conserved Genomic Sequence With Rearrangements.” Genome Research 14: 1394–1403. 15231754 10.1101/gr.2289704 PMC442156

[ece371568-bib-0010] Darriba, D. , G. L. Taboada , R. Doallo , and D. Posada . 2012. “jModelTest 2: More Models, New Heuristics and Parallel Computing.” Nature Methods 9: 772. 10.1038/nmeth.2109 PMC4594756 22847109

[ece371568-bib-0011] Dierckxsens, N. , P. Mardulyn , and G. Smits . 2017. “NOVOPlasty: de Novo Assembly of Organelle Genomes From Whole Genome Data.” Nucleic Acids Research 45: e18. 28204566 10.1093/nar/gkw955 PMC5389512

[ece371568-bib-0012] Drescher, A. , S. Ruf , T. Calsa Jr. , H. Carrer , and R. J. T. P. J. Bock . 2000. “The Two Largest Chloroplast Genome‐Encoded Open Reading Frames of Higher Plants Are Essential Genes.” Plant Journal 22, no. 2: 97–104. 10.1046/j.1365-313x.2000.00722.x 10792825

[ece371568-bib-0013] Drouin, G. , H. Daoud , and J. Xia . 2008. “Relative Rates of Synonymous Substitutions in the Mitochondrial, Chloroplast and Nuclear Genomes of Seed Plants.” Molecular Phylogenetics and Evolution 49, no. 3: 827–831. 18838124 10.1016/j.ympev.2008.09.009

[ece371568-bib-0014] Du, Z. , F. Yuan , Y. Huang , et al. 2022. “The Complete Chloroplast Genome of *Salvia liguliloba* Y. Z. Sun (Lamiaceae).” Mitochondrial DNA Part B Resources 7: 1355–1356. 35903301 10.1080/23802359.2022.2097486 PMC9318229

[ece371568-bib-0015] Ebrahimi‐Fallah, A. , and H. Askari . 2024. “Regardless of having identical photosynthetic pathways, chloroplast genomes vary depending on whether the host plant is monocotyledonous or dicotyledonous.” Genetic Resources and Crop Evolution 71: 3583–3602.

[ece371568-bib-0016] Feng, J. Y. , X. J. Jin , S. L. Zhang , et al. 2022. “ *Smilax weniae*, a New Species of Smilacaceae From Limestone Areas Bordering Guizhou and Guangxi, China.” Plants 11, no. 8: 1032. 35448760 10.3390/plants11081032 PMC9028124

[ece371568-bib-0017] Fernandes, D. C. , B. P. Martins , G. P. da Silva , et al. 2022. “ *Echinodorus macrophyllus* Fraction With a High Level of Flavonoid Inhibits Peripheral and Central Mechanisms of Nociception.” Journal of Traditional and Complementary Medicine 12: 123–130. 35528477 10.1016/j.jtcme.2021.07.001 PMC9072821

[ece371568-bib-0018] Ferreira, M. I. , G. G. Gonçalves , and L. C. Ming . 2018. “ *Echinodorus macrophyllus* (Kunth) Micheli.” Medicinal and Aromatic Plants of the World 5: 211–217.

[ece371568-bib-0019] Frazer, K. A. , L. Pachter , A. Poliakov , E. M. Rubin , and I. Dubchak . 2004. “VISTA: Computational Tools for Comparative Genomics.” Nucleic Acids Research 32: W273–W279. 15215394 10.1093/nar/gkh458 PMC441596

[ece371568-bib-0020] Geng, X. S. , N. Huang , Y. Zhu , L. Qin , and L. Hui . 2022. “Codon Usage Bias Analysis of the Chloroplast Genome of Cassava.” South African Journal of Botany 151: 970–975.

[ece371568-bib-0021] Govaerts, R. , E. Nic Lughadha , N. Black , R. Turner , and A. Paton . 2021. “The World Checklist of Vascular Plants, a Continuously Updated Resource for Exploring Global Plant Diversity.” Scientific Data 8: 215. 34389730 10.1038/s41597-021-00997-6 PMC8363670

[ece371568-bib-0022] Guan, B. , J. Wen , H. Guo , and Y. Liu . 2024. “Comparative and Phylogenetic Analyses Based on the Complete Chloroplast Genome of *Cornus* Subg. *Syncarpea* (Cornaceae) Species.” Frontiers in Plant Science 15: 1306196. 38545387 10.3389/fpls.2024.1306196 PMC10965615

[ece371568-bib-0023] Guo, H. , L. Wang , W. Xu , et al. 2022. “The Complete Chloroplast Genome Sequence of *Cyathula officinalis* and Comparative Analysis With Four Related Species.” Gene 839: 146728. 35850203 10.1016/j.gene.2022.146728

[ece371568-bib-0024] Guo, L. , X. Wang , R. H. Wang , and P. Li . 2023. “Characterization and Comparative Analysis of Chloroplast Genomes of Medicinal Herb *Scrophularia ningpoensis* and Its Common Adulterants (Scrophulariaceae).” International Journal of Molecular Sciences 24: 10034. 37373180 10.3390/ijms241210034 PMC10298345

[ece371568-bib-0025] Handajani, H. , W. Widanarni , T. Budiardi , M. Setiawati , and S. Sujono . 2018. “Phytoremediation of Eel (*Anguilla bicolor* Bicolor) Rearing Wastewater Using Amazon Sword (*Echinodorus amazonicus*) and Water Jasmine (*Echinodorus palaefolius*).” Omni‐Akuatika 14: 43–51.

[ece371568-bib-0026] Hong, Z. , Z. Wu , K. Zhao , et al. 2020. “Comparative Analyses of Five Complete Chloroplast Genomes From the Genus *Pterocarpus* (Fabacaeae).” International Journal of Molecular Sciences 21: 3758. 32466556 10.3390/ijms21113758 PMC7312355

[ece371568-bib-0027] Ito, Y. , and N. Tanaka . 2023. “Phylogeny of *Alisma* (Alismataceae) Revisited: Implications for Polyploid Evolution and Species Delimitation.” Journal of Plant Research 136: 613–629. 37402089 10.1007/s10265-023-01477-1

[ece371568-bib-0028] Ivanova, Z. , G. Sablok , E. Daskalova , et al. 2017. “Chloroplast Genome Analysis of Resurrection Tertiary Relict *Haberlea rhodopensis* Highlights Genes Important for Desiccation Stress Response.” Frontiers in Plant Science 8: 204. 28265281 10.3389/fpls.2017.00204 PMC5316520

[ece371568-bib-0029] Jansen, R. K. , Z. Cai , L. A. Raubeson , et al. 2007. “Analysis of 81 Genes From 64 Plastid Genomes Resolves Relationships in Angiosperms and Identifies Genome‐Scale Evolutionary Patterns.” Proceedings of the National Academy of Sciences of the United States of America 104: 19369–19374. 18048330 10.1073/pnas.0709121104 PMC2148296

[ece371568-bib-0030] Jin, J. J. , W. B. Yu , J. B. Yang , et al. 2020. “GetOrganelle: A Fast and Versatile Toolkit for Accurate de Novo Assembly of Organelle Genomes.” Genome Biology 21: 241. 32912315 10.1186/s13059-020-02154-5 PMC7488116

[ece371568-bib-0031] Katoh, K. , and D. M. Standley . 2013. “MAFFT Multiple Sequence Alignment Software Version 7: Improvements in Performance and Usability.” Molecular Biology and Evolution 30: 772–780. 23329690 10.1093/molbev/mst010 PMC3603318

[ece371568-bib-0032] Kearse, M. , R. Moir , A. Wilson , et al. 2012. “Geneious Basic: An Integrated and Extendable Desktop Software Platform for the Organization and Analysis of Sequence Data.” Bioinformatics 28: 1647–1649. 22543367 10.1093/bioinformatics/bts199 PMC3371832

[ece371568-bib-0033] Kim, K. J. , K. S. Choi , and R. K. Jansen . 2005. “Two Chloroplast DNA Inversions Originated Simultaneously During the Early Evolution of the Sunflower Family (Asteraceae).” Molecular Biology and Evolution 22: 1783–1792. 15917497 10.1093/molbev/msi174

[ece371568-bib-0034] Kurtz, S. , J. V. Choudhuri , E. Ohlebusch , C. Schleiermacher , J. Stoye , and R. Giegerich . 2001. “REPuter: The Manifold Applications of Repeat Analysis on a Genomic Scale.” Nucleic Acids Research 29: 4633–4642. 11713313 10.1093/nar/29.22.4633 PMC92531

[ece371568-bib-0035] Lan, Z. Q. , W. Zheng , A. Talavera , et al. 2024. “Comparative and Phylogenetic Analyses of Plastid Genomes of the Medicinally Important Genus *Alisma* (Alismataceae).” Frontiers in Plant Science 15: 1415253. 39233910 10.3389/fpls.2024.1415253 PMC11372848

[ece371568-bib-0036] Lee, C. , T. A. Ruhlman , and R. K. Jansen . 2020. “Unprecedented Intraindividual Structural Heteroplasmy in *Eleocharis* (Cyperaceae, Poales) Plastomes.” Genome Biology and Evolution 12: 641–655. 32282915 10.1093/gbe/evaa076 PMC7426004

[ece371568-bib-0037] Lehtonen, S. 2006. “Phylogenetics of *Echinodorus* (Alismataceae) Based on Morphological Data.” Botanical Journal of the Linnean Society 150: 291–305.

[ece371568-bib-0038] Lehtonen, S. 2017. “Splitting *Caldesia* in Favour of *Albidella* (Alismataceae).” Australian Systematic Botany 30: 64–69.

[ece371568-bib-0039] Lehtonen, S. , and L. Myllys . 2008. “Cladistic Analysis of *Echinodorus* (Alismataceae): Simultaneous Analysis of Molecular and Morphological Data.” Cladistics 24: 218–239.

[ece371568-bib-0040] Lehwark, P. , and S. Greiner . 2019. “GB2sequin – A File Converter Preparing Custom GenBank Files for Database Submission.” Genomics 111, no. 4: 759–761. 29842948 10.1016/j.ygeno.2018.05.003

[ece371568-bib-0041] Les, D. H. , D. K. Garvin , and C. F. Wimpee . 1991. “Molecular Evolutionary History of Ancient Aquatic Angiosperms.” Proceedings of the National Academy of Sciences, USA 88: 10119–10123. 10.1073/pnas.88.22.10119 PMC52879 1946432

[ece371568-bib-0042] Li, D. M. , Y. G. Pan , H. L. Liu , B. Yu , D. Huang , and G. F. Zhu . 2024. “Thirteen Complete Chloroplast Genomes of the Costaceae Family: Insights Into Genome Structure, Selective Pressure and Phylogenetic Relationships.” BMC Genomics 25: 68. 38233753 10.1186/s12864-024-09996-4 PMC10792896

[ece371568-bib-0043] Li, H. , Q. Guo , L. Xu , H. Gao , L. Liu , and X. Zhou . 2023. “CPJSdraw: Analysis and Visualization of Junction Sites of Chloroplast Genomes.” Peer Journal 11: e15326. 10.7717/peerj.15326 PMC10182761 37193025

[ece371568-bib-0044] Li, Z. Z. , S. Lehtonen , and J. M. Chen . 2023. “The Dynamic History of Plastome Structure Across Aquatic Subclass Alismatidae.” BMC Plant Biology 23: 125. 36869282 10.1186/s12870-023-04125-x PMC9985265

[ece371568-bib-0045] Li, Z. Z. , S. Lehtonen , K. Martins , Q. F. Wang , and J. M. Chen . 2022. “Complete Genus‐Level Plastid Phylogenomics of Alismataceae With Revisited Historical Biogeography.” Molecular Phylogenetics and Evolution 166: 107334. 34715331 10.1016/j.ympev.2021.107334

[ece371568-bib-0046] Liang, J. , Q. Ma , and Z. Yang . 2019. “The First Complete Chloroplast Genomes of Two Alismataceae Species, and the Phylogenetic Relationship Under Order Alismatales.” Mitochondrial DNA Part B Resources 4, no. 1: 122–123.

[ece371568-bib-0047] Liao, Y. Y. , Y. Liu , X. Liu , et al. 2020. “The Complete Chloroplast Genome of *Myriophyllum spicatum* Reveals a 4‐kb Inversion and New Insights Regarding Plastome Evolution in Haloragaceae.” Ecology and Evolution 10: 3090–3102. 32211179 10.1002/ece3.6125 PMC7083656

[ece371568-bib-0048] Librado, P. , and J. Rozas . 2009. “DnaSP v5: A Software for Comprehensive Analysis of DNA Polymorphism Data.” Bioinformatics 25: 1451–1452. 19346325 10.1093/bioinformatics/btp187

[ece371568-bib-0049] Liu, E. , C. Yang , J. Liu , et al. 2019. “Comparative Analysis of Complete Chloroplast Genome Sequences of Four Major *Amorphophallus* Species.” Scientific Reports 9: 809. 30692573 10.1038/s41598-018-37456-z PMC6349887

[ece371568-bib-0050] Liu, S. , Y. Ni , J. Li , et al. 2023. “CPGView: A Package for Visualizing Detailed Chloroplast Genome Structures.” Molecular Ecology Resources 23, no. 3: 694–704. 36587992 10.1111/1755-0998.13729

[ece371568-bib-0051] Liu, F. , Y. W. Zhang , X. H. Cui , J. M. Chen , and Q. F. Wang . 2009. “ *Sagittaria lichuanensis*—A Newly Recorded Species in Alismataceae From Guizhou Province.” Journal of Tropical and Subtropical Botany 1: 90–92.

[ece371568-bib-0052] Luo, Y. , P. F. Ma , H. T. Li , J. B. Yang , H. Wang , and D. Z. Li . 2016. “Plastid Phylogenomic Analyses Resolve Tofieldiaceae as the Root of the Early Diverging Monocot Order Alismatales.” Genome Biology and Evolution 8: 932–945. 26957030 10.1093/gbe/evv260 PMC4823975

[ece371568-bib-0053] Matias, L. Q. 2007. “O gênero *Echinodorus* (Alismataceae) do domínio da *Caatinga brasileira* .” Rodriguésia 58: 743–774.

[ece371568-bib-0054] Matias, L. Q. 2010. “A Synopsis of Alismataceae From the Semi‐Arid Region of Northeastern Brazil.” Revista Caatinga 23: 46–53.

[ece371568-bib-0055] Miller, M. A. , W. Pfeiffer , and T. Schwartz . 2010. Creating the CIPRES Science Gateway for Inference of Large Phylogenetic Trees. 2010 Gateway Computing Environments Workshop (GCE), 1–8.

[ece371568-bib-0056] Mwanzia, V. M. , J. M. Nzei , D. Y. Yan , P. W. Kamau , J. M. Chen , and Z. Z. Li . 2019. “The Complete Chloroplast Genomes of Two Species in Threatened Monocot Genus *Caldesia* in China.” Genetica 147, no. 5–6: 381–390. 31654262 10.1007/s10709-019-00079-x

[ece371568-bib-0057] Nguyen, L. T. , H. A. Schmidt , A. von Haeseler , and B. Q. Minh . 2015. “IQ‐TREE: A Fast and Effective Stochastic Algorithm for Estimating Maximum‐Likelihood Phylogenies.” Molecular Biology and Evolution 32: 268–274. 25371430 10.1093/molbev/msu300 PMC4271533

[ece371568-bib-0058] Plants of the World Online (POWO) . 2023. Facilitated by the Royal Botanic Gardens, Kew. http://www.plantsoftheworldonline.org/.

[ece371568-bib-0059] Rataj, K. 1970. “Three New Species of the Genus *Echinodorus* Imported for the Decoration of Aquaria.” Preslia 42: 264–266.

[ece371568-bib-0060] Rice, P. , I. Longden , and A. Bleasby . 2000. “EMBOSS: The European Molecular Biology Open Software Suite.” Trends in Genetics 16: 276–277. 10827456 10.1016/s0168-9525(00)02024-2

[ece371568-bib-0061] Ross, T. G. , C. F. Barrett , M. Soto Gomez , et al. 2016. “Plastid Phylogenomics and Molecular Evolution of Alismatales.” Cladistics 32: 160–178. 34736309 10.1111/cla.12133

[ece371568-bib-0062] Shahzadi, I. , A. Abdullah , F. Mehmood , Z. Ali , I. Ahmed , and B. Mirza . 2020. “Chloroplast Genome Sequences of *Artemisia maritima* and *Artemisia absinthium*: Comparative Analyses, Mutational Hotspots in Genus *Artemisia* and Phylogeny in Family Asteraceae.” Genomics 112: 1454–1463. 31450007 10.1016/j.ygeno.2019.08.016

[ece371568-bib-0063] Shi, W. , S. Hu , W. Song , Y. Huang , C. Shi , and S. Wang . 2023. “Uncovering the First Complete Chloroplast Genomics, Comparative Analysis, and Phylogenetic Relationships of the Medicinal Plants *Rhamnus cathartica* and *Frangula alnus* (Rhamnaceae).” Physiology and Molecular Biology of Plants 29: 855–869. 37520808 10.1007/s12298-023-01331-7 PMC10382440

[ece371568-bib-0064] Shi, W. , Y. Huang , S. Hu , et al. 2024. “Exploring the Chloroplast Genomics, Comparative Analysis, Evolution, and Phylogenetic Relationships of *Phylica pubescens* (Rhamnaceae) in the Cape Flora.” South African Journal of Botany 164: 374–385.

[ece371568-bib-0065] Shi, W. B. , W. L. Shi , W. C. Song , Y. Q. Zhao , C. Shi , and S. Wang . 2023. “Complete Chloroplast Genomes of Four *Atalantia* (Rutaceae) Species: Insights Into Comparative Analysis, Phylogenetic Relationships, and Divergence Time Estimation.” Plant Systematics and Evolution 5: 309.

[ece371568-bib-0066] da Silva, G. P. , D. C. Fernandes , W. S. Pereira , et al. 2024. “ *Echinodorus macrophyllus*: Acute Toxicological Evaluation of Hydroxycinnamoyl Derivatives From SF1 Subfractions.” Journal of Ethnopharmacology 321: 117476. 38008274 10.1016/j.jep.2023.117476

[ece371568-bib-0067] Silva, S. R. , Y. C. Diaz , H. A. Penha , et al. 2016. “The Chloroplast Genome of *Utricularia reniformis* Sheds Light on the Evolution of the ndh Gene Complex of Terrestrial Carnivorous Plants From the Lentibulariaceae Family.” PLoS One 11, no. 10: e0165176. 27764252 10.1371/journal.pone.0165176 PMC5072713

[ece371568-bib-0068] Silva, S. R. , D. G. Pinheiro , H. A. Penha , et al. 2019. “Intraspecific Variation Within the *Utricularia amethystina* Species Morphotypes Based on Chloroplast Genomes.” International Journal of Molecular Sciences 20: 6130. 31817365 10.3390/ijms20246130 PMC6940893

[ece371568-bib-0069] da Silva, Y. J. A. B. , J. R. B. Cantalice , V. P. Singh , C. M. C. A. Cruz , and W. L. da Silva Souza . 2016. “Sediment Transport Under the Presence and Absence of Emergent Vegetation in a Natural Alluvial Channel From Brazil.” International Journal of Sediment Research 31: 360–367.

[ece371568-bib-0070] Song, Y. X. , M. Shen , F. L. Cao , and X. M. Yang . 2024. “Compare Analysis of Codon Usage Bias of Nuclear Genome in Eight Sapindaceae Species.” International Journal of Molecular Sciences 26, no. 1: 39. 39795897 10.3390/ijms26010039 PMC11720230

[ece371568-bib-0071] Stant, M. Y. 1964. “Anatomy of the Alismataceae.” Journal of the Linnean Society of London, Botany 59, no. 376: 1–42.

[ece371568-bib-0072] Tanus‐Rangel, E. , S. R. Santos , J. C. Lima , et al. 2010. “Topical and Systemic Anti‐Inflammatory Effects of *Echinodorus macrophyllus* (Kunth) Micheli (Alismataceae).” Journal of Medicinal Food 13: 1161–1166. 20828306 10.1089/jmf.2009.0247

[ece371568-bib-0073] The Angiosperm Phylogeny Group . 2016. “An Update of the Angiosperm Phylogeny Group Classification for the Orders and Families of Flowering Plants: APG IV.” Botanical Journal of the Linnean Society 181, no. 1: 1–20.

[ece371568-bib-0074] Tillich, M. , P. Lehwark , T. Pellizzer , et al. 2017. “GeSeq – Versatile and Accurate Annotation of Organelle Genomes.” Nucleic Acids Research 45: W6–W11. 28486635 10.1093/nar/gkx391 PMC5570176

[ece371568-bib-0075] Wang, D. , Y. Zhang , Z. Zhang , J. Zhu , and J. Yu . 2010. “KaKs_Calculator 2.0: A Toolkit Incorporating Gamma‐Series Methods and Sliding Window Strategies.” Genomics, Proteomics & Bioinformatics 8: 77–80. 10.1016/S1672-0229(10)60008-3 PMC5054116 20451164

[ece371568-bib-0076] Wang, J. , S. Kan , X. Liao , et al. 2024. “Plant Organellar Genomes: Much Done, Much More to Do.” Trends in Plant Science 29: 754–769. 38220520 10.1016/j.tplants.2023.12.014

[ece371568-bib-0077] Wang, L. , Z. Huo , W. Xu , et al. 2024. “Comparative Plastomes of Eight Subgenus *Chamaesyce* Plants and System Authentication of Euphorbiae Humifusae Herba.” Food Chemistry 447: 139039. 38518619 10.1016/j.foodchem.2024.139039

[ece371568-bib-0078] Wang, R. H. , M. M. Chen , Y. Z. Wu , et al. 2021. “The Complete Chloroplast Genome Sequence of Wild Japanese Pepper *Tubocapsicum anomalum* Makino (Solanaceae).” Mitochondrial DNA Part B Resources 6: 2322–2323. 34345686 10.1080/23802359.2021.1950063 PMC8284131

[ece371568-bib-0079] Wang, R. H. , J. Gao , J. Y. Feng , et al. 2022. “Comparative and Phylogenetic Analyses of Complete Chloroplast Genomes of *Scrophularia incisa* Complex (Scrophulariaceae).” Genes 13, no. 10: 1691. 36292576 10.3390/genes13101691 PMC9601301

[ece371568-bib-0080] Wang, Y. H. , S. Wicke , H. Wang , et al. 2018. “Plastid Genome Evolution in the Early‐Diverging Legume Subfamily Cercidoideae (Fabaceae).” Frontiers in Plant Science 9: 138. 29479365 10.3389/fpls.2018.00138 PMC5812350

[ece371568-bib-0081] Wanichthanarak, K. , I. Nookaew , P. Pasookhush , et al. 2023. “Revisiting Chloroplast Genomic Landscape and Annotation Towards Comparative Chloroplast Genomes of Rhamnaceae.” BMC Plant Biology 23: 59. 36707785 10.1186/s12870-023-04074-5 PMC9883906

[ece371568-bib-0082] Weng, L. , Y. Jiang , Y. Wang , et al. 2023. “Chloroplast Genome Characteristics and Phylogeny of the *Sinodielsia* Clade (Apiaceae: Apioideae).” BMC Plant Biology 23: 284. 37246219 10.1186/s12870-023-04271-2 PMC10226202

[ece371568-bib-0083] Wick, R. R. , M. B. Schultz , J. Zobel , and K. E. Holt . 2015. “Bandage: Interactive Visualization of de Novo Genome Assemblies.” Bioinformatics 31: 3350–3352. 26099265 10.1093/bioinformatics/btv383 PMC4595904

[ece371568-bib-0084] Wu, H. , D. Z. Li , and P. F. Ma . 2024. “Unprecedented Variation Pattern of Plastid Genomes and the Potential Role in Adaptive Evolution in Poales.” BMC Biology 22: 97. 38679718 10.1186/s12915-024-01890-5 PMC11057118

[ece371568-bib-0085] Wu, L. , P. Fan , J. Cai , et al. 2024. “Comparative Genomics and Phylogenomics of the Genus *Glycyrrhiza* (Fabaceae) Based on Chloroplast Genomes.” Frontiers in Pharmacology 15: 1371390. 38515836 10.3389/fphar.2024.1371390 PMC10955637

[ece371568-bib-0086] Wu, Y. , F. Liu , D. G. Yang , et al. 2018. “Comparative Chloroplast Genomics of *Gossypium* Species: Insights Into Repeat Sequence Variations and Phylogeny.” Frontiers in Plant Science 9: 376. 29619041 10.3389/fpls.2018.00376 PMC5871733

[ece371568-bib-0087] Xiang, Y. N. , X. Q. Wang , L. L. Ding , et al. 2024. “Deciphering the Plastomic Code of Chinese Hog‐Peanut (*Amphicarpaea edgeworthii* Benth., Leguminosae): Comparative Genomics and Evolutionary Insights Within the Phaseoleae Tribe.” Genes 15, no. 1: 88. 38254977 10.3390/genes15010088 PMC10815570

[ece371568-bib-0088] Xie, P. , L. Tang , Y. Luo , C. Liu , and H. Yan . 2023. “Plastid Phylogenomic Insights Into the Inter‐Tribal Relationships of Plantaginaceae.” Biology‐Basel 12: 263. 36829541 10.3390/biology12020263 PMC9953724

[ece371568-bib-0089] Xu, C. , X. Cai , Q. Chen , H. Zhou , Y. Cai , and A. Ben . 2011. “Factors Affecting Synonymous Codon Usage Bias in Chloroplast Genome of Oncidium Gower Ramsey.” Evolutionary Bioinformatics 7: 271–278. 22253533 10.4137/EBO.S8092 PMC3255522

[ece371568-bib-0090] Xu, Z. , T. Xin , D. Bartels , et al. 2018. “Genome Analysis of the Ancient Tracheophyte *selaginella Tamariscina* Reveals Evolutionary Features Relevant to the Acquisition of Desiccation Tolerance.” Molecular Plant 11: 983–994. 29777775 10.1016/j.molp.2018.05.003

[ece371568-bib-0091] Yin, D. P. , B. Pang , H. B. Li , et al. 2022. “The Complete Chloroplast Genome of the Medical Plant *Huperzia crispata* From the Huperziaceae Family: Structure, Comparative Analysis, and Phylogenetic Relationships.” Molecular Biology Reports 49, no. 12: 11729–11741. 36197623 10.1007/s11033-022-07979-w

[ece371568-bib-0092] Zeng, W. Q. , H. J. Yan , Y. Z. Wu , et al. 2021. “The Complete Chloroplast Genome Sequence of Japanese Buttercup *Ranunculus Japonicus* Thunb.” Mitochondrial DNA Part B Resources 6, no. 11: 3186–3187. 34660897 10.1080/23802359.2021.1987166 PMC8519513

[ece371568-bib-0093] Zhang, M. , and N. Chen . 2022. “Comparative Analysis of *Thalassionema* Chloroplast Genomes Revealed Hidden Biodiversity.” BMC Genomics 23, no. 1: 327. 35477350 10.1186/s12864-022-08532-6 PMC9044688

[ece371568-bib-0094] Zhang, Z. , Y. Jin , Y. Gao , et al. 2023. “The Complete Chloroplast Genomes of Two *Physalis* Species, *Physalis macrophysa* and *P. ixocarpa*: Comparative Genomics, Evolutionary Dynamics and Phylogenetic Relationships.” Agronomy 13, no. 1: 135.

[ece371568-bib-0095] Zheng, W. , J. Liu , W. Zhao , Z. Zhao , Z. Lan , and J. Wen . 2024. “The Complete Chloroplast Genomes of Three Alismataceae Species, Including the Medicinally Important *Alisma orientale* .” Mitochondrial DNA Part B Resources 9: 385–389. 38562439 10.1080/23802359.2024.2320419 PMC10984228

[ece371568-bib-0096] Zhou, Z. , J. Wang , T. Pu , et al. 2022. “Comparative Analysis of Medicinal Plant *Isodon rubescens* and Its Common Adulterants Based on Chloroplast Genome Sequencing.” Frontiers in Plant Science 13: 1036277. 36479509 10.3389/fpls.2022.1036277 PMC9720329

[ece371568-bib-0097] Zhu, J. Q. , Y. Huang , W. G. Chai , and P. G. Xia . 2024. “Decoding the Chloroplast Genome of *Tetrastigma* (Vitaceae): Variations and Phylogenetic Selection Insights.” International Journal of Molecular Sciences 25, no. 15: 8290. 39125860 10.3390/ijms25158290 PMC11312916

