# Supplementary figures and images for: Comparative Genomics and Phylogenetic Analyses of Aquarius macrophyllus and Related Genera in Alismataceae Based on Plastome Data
Source: Ecol Evol. 2025 Jun 10;15(6):e71568. doi: 10.1002/ece3.71568 (PMC12152199; doi:10.1002/ece3.71568)

Cis-splicing Genes

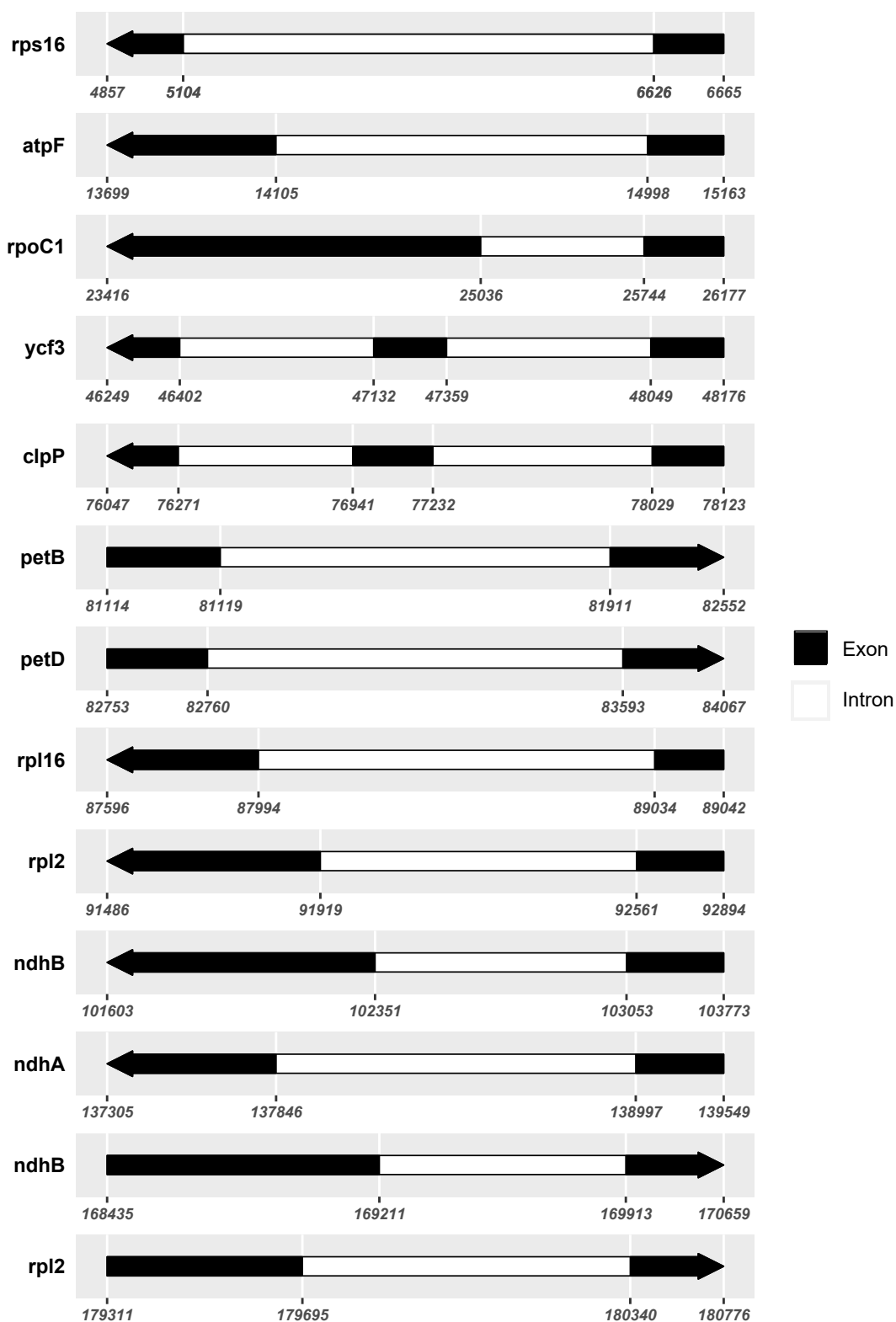

Figure S1. Exon and intron structure mapping of cis-spliced genes.

Supplement: Supplementary file 1 — Figure S1. Exon and intron structure mapping of cis‐spliced genes. [file ECE3-15-e71568-s004.pdf]

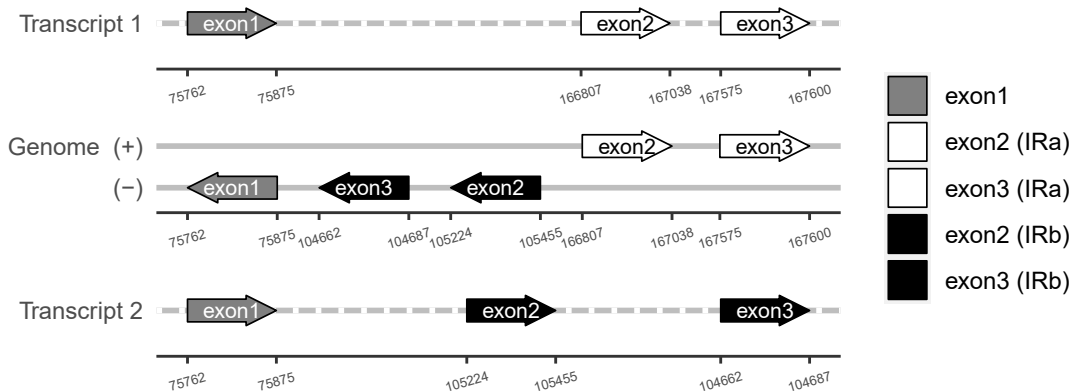

Figure S2. Detailed structure of the trans-spliced gene *rps12*.

Supplement: Supplementary file 2 — Figure S2. Detailed structure of the trans‐spliced gene rps12. [file ECE3-15-e71568-s002.pdf]

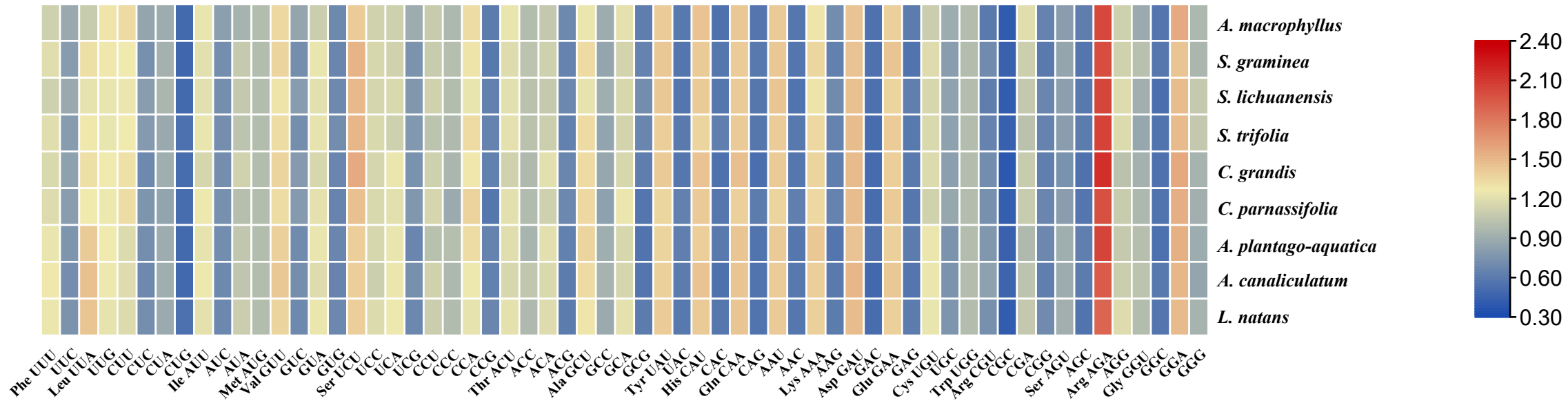

Figure S4. RSCU values heatmap of the codons in the nine Alismataceae plastomes.

Supplement: Supplementary file 4 — Figure S4. RSCU values heatmap of the codons in the nine Alismataceae plastomes. [file ECE3-15-e71568-s010.pdf]
