# Supplementary material for: Comparative Genomics and Phylogenetic Analyses of Aquarius macrophyllus and Related Genera in Alismataceae Based on Plastome Data
Source: Ecol Evol. 2025 Jun 10;15(6):e71568. doi: 10.1002/ece3.71568 (PMC12152199; doi:10.1002/ece3.71568)
Supplement: Supplementary file 3 — Figure S3. Synteny and rearrangements detected in nine plastomes. Color bars indicate syntenic blocks and connecting lines indicate corresponding blocks. [file ECE3-15-e71568-s009.pdf]

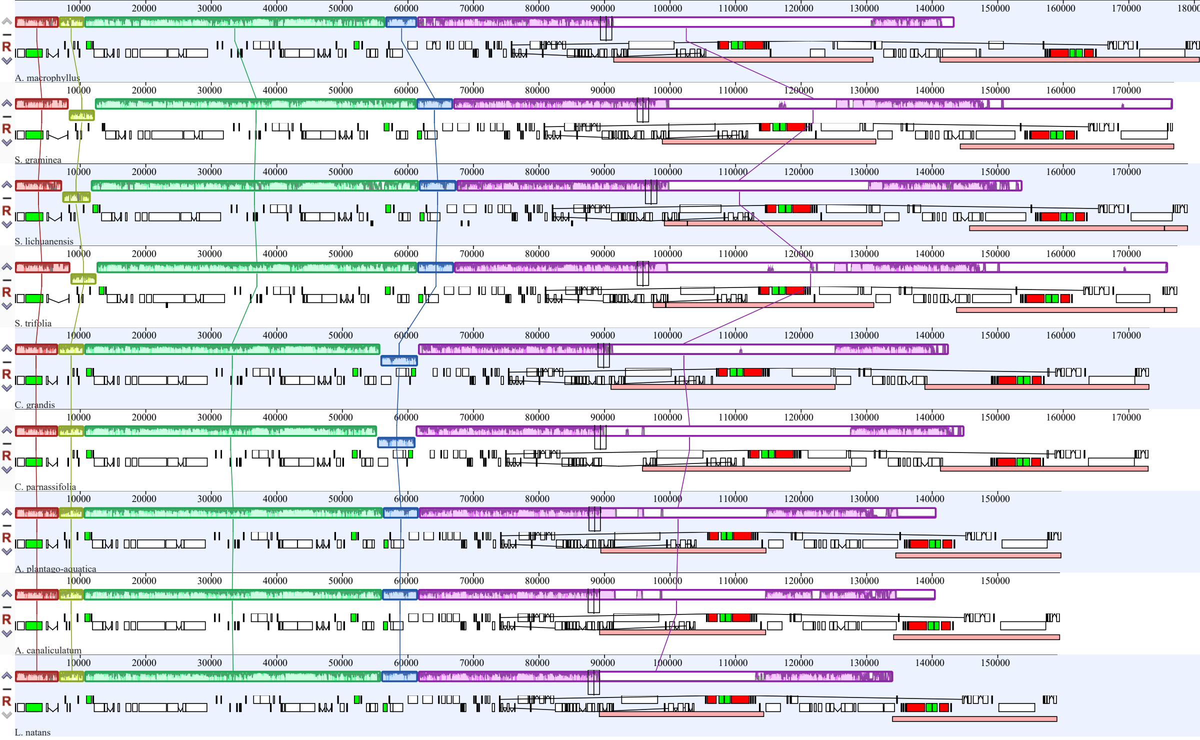

Figure S3. Synteny and rearrangements detected in 9 plastomes. Color bars indicate syntenic blocks and connecting lines indicate corresponding blocks.
