# Supplementary material for: Comparative Genomics and Phylogenetic Analyses of Aquarius macrophyllus and Related Genera in Alismataceae Based on Plastome Data
Source: Ecol Evol. 2025 Jun 10;15(6):e71568. doi: 10.1002/ece3.71568 (PMC12152199; doi:10.1002/ece3.71568)
Supplement: Supplementary file 5 — Table S1. Coding genes of the Aquarius macrophyllus plastome. [file ECE3-15-e71568-s006.docx]

**Table S1.** Coding genes of the *Aquarius macrophyllus* plastome*.*

| Category | Gene group | Gene name |
| --- | --- | --- |
| Photosynthesis | Subunits of photosystem I | *psaA*, *psaB*, *psaC*, *psaI*, *psaJ* |
|  | Subunits of photosystem II | *psbA, psbB, psbC, psbD, psbE, psbF, psbH, psbI, psbJ, psbK, psbL, psbM, psbN, psbT, psbZ* |
|  | Subunits of NADH dehydrogenase | *ndhA*, ndhB**(2)*, ndhC, ndhD, ndhE, ndhF*(2), ndhG*, ndhH, ndhI, ndhJ, ndhK* |
|  | Subunits of cytochrome b/f complex | *petA, petB**, *petD**, *petG, petL, petN* |
|  | Subunits of ATP synthase | *atpA, atpB, atpE, atpF**, *atpH, atpI* |
|  | Large subunit of rubisco | *rbcL* |
| Self-replication | Proteins of large ribosomal subunit | *rpl14, rpl16*, rpl2**(2)*, rpl20, rpl22, rpl23*(2)*, rpl32*(2)*, rpl33, rpl3*6 |
|  | Proteins of small ribosomal subunit | *rps11, rps12***(2)*, rps14, rps15, rps16*, rps18, rps19, rps2, rps3, rps4, rps7*(2)*, rps8* |
|  | Subunits of RNA polymerase | *rpoA, rpoB, rpoC1**, *rpoC2* |
|  | Ribosomal RNAs | *rrn16S*(2)*, rrn23S*(2)*, rrn4.5S*(2)*, rrn5S*(2) |
|  | Transfer RNAs | *trnA-UGC**(2)*, trnC-GCA, trnD-GUC, trnE-UUC, trnF-GAA, trnfM-CAU, trnG-GCC, trnG-UCC*, trnH-GUG, trnI-CAU*(2)*, trnI-GAU**(2)*, trnK-UUU*, trnL-CAA*(2)*, trnL-UAA*, trnL-UAG, trnM-CAU, trnN-GUU*(2)*, trnP-UGG, trnQ-UUG, trnR-ACG*(2)*, trnR-UCU, trnS-GCU, trnS-GGA, trnS-UGA, trnT-GGU, trnT-UGU, trnV-GAC*(2)*, trnV-UAC, trnW-CCA, trnY-GUA* |
| Other genes | Maturase | *matK* |
|  | Protease | *clpP*** |
|  | Envelope membrane protein | *cemA* |
|  | c-type cytochrome synthesis gene | *ccsA* |
|  | Subunit of Acetyl-CoA-carboxylase | *accD* |
|  | Translational initiation factor | *infA* |
|  | other | - |
| Genes of unknown function | Conserved hypothetical chloroplast ORF | *ycf1*(2)*, ycf2*(2)*, ycf3***, *ycf4* |

Notes: Gene *: Gene with one intron; Gene **: Gene with two introns; Gene (2): Number of copies of multi-copy genes.
